# Matching electron transport layers with a non-halogenated and low synthetic complexity polymer:fullerene blend for efficient outdoor and indoor organic photovoltaics†

**DOI:** 10.1039/d2ta01205g

**Published:** 2022-04-19

**Authors:** Xabier Rodríguez-Martínez, Sergi Riera-Galindo, Jiayan Cong, Thomas Österberg, Mariano Campoy-Quiles, Olle Inganäs

**Affiliations:** Biomolecular and Organic Electronics, Department of Physics, Chemistry and Biology, Linköping University Linköping 58183 Sweden sergi.riera-galindo@liu.se; Epishine AB Wahlbecksgatan 25 Linköping 58213 Sweden; Instituto de Ciencia de Materiales de Barcelona, ICMAB-CSIC Campus UAB Bellaterra 08193 Spain

## Abstract

The desired attributes of organic photovoltaics (OPV) as a low cost and sustainable energy harvesting technology demand the use of non-halogenated solvent processing for the photoactive layer (PAL) materials, preferably of low synthetic complexity (SC) and without compromising the power conversion efficiency (PCE). Despite their record PCEs, most donor–acceptor conjugated copolymers in combination with non-fullerene acceptors are still far from upscaling due to their high cost and SC. Here we present a non-halogenated and low SC ink formulation for the PAL of organic solar cells, comprising PTQ10 and PC_61_BM as donor and acceptor materials, respectively, showing a record PCE of 7.5% in blade coated devices under 1 sun, and 19.9% under indoor LED conditions. We further study the compatibility of the PAL with 5 different electron transport layers (ETLs) in inverted architecture. We identify that commercial ZnO-based formulations together with a methanol-based polyethyleneimine-Zn (PEI-Zn) chelated ETL ink are the most suitable interlayers for outdoor conditions, providing fill factors as high as 74% and excellent thickness tolerance (up to 150 nm for the ETL, and >200 nm for the PAL). In indoor environments, SnO_2_ shows superior performance as it does not require UV photoactivation. Semi-transparent devices manufactured entirely in air *via* lamination show indoor PCEs exceeding 10% while retaining more than 80% of the initial performance after 400 and 350 hours of thermal and light stress, respectively. As a result, PTQ10:PC_61_BM combined with either PEI-Zn or SnO_2_ is currently positioned as a promising system for industrialisation of low cost, multipurpose OPV modules.

## Abstract 2

A non-halogenated and low synthetic complexity ink formulation for the photoactive layer of organic solar cells, showing a record PCE of 7.5% in blade coated devices under 1 sun and 19.9% under indoor LED illumination.

## Graphical abstract

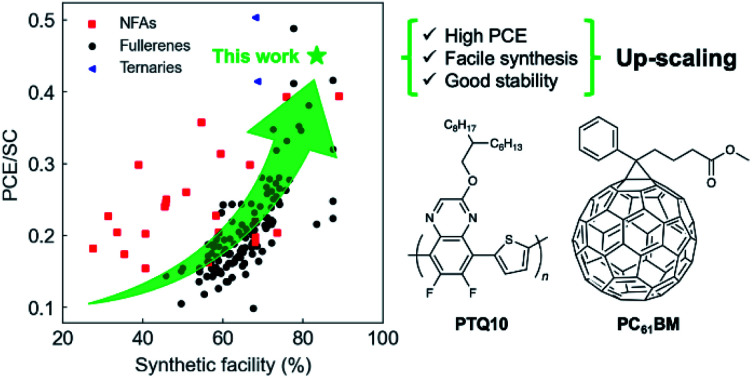

## Introduction

The latest advances in the organic photovoltaics (OPV) field have boosted power conversion efficiency (PCE) records in lab scale very close to the 20% milestone in single-junction devices under 1 sun.^1^ Under indoor lighting conditions, OPV finds its ideal application niche on myriad flexible, lightweight and form factor devices, primarily as off-grid energy sources in small and portable internet of things (IoT) items. Indoor OPV devices show PCE records approaching 30% (ref. 2 and 3) depending on the light emission spectrum (warm/cold light-emitting diodes (LEDs) or fluorescent lighting) and intensity (50–2000 lux), which broadly outperform the figures provided by competing technologies such as crystalline silicon.^4^ The promised unique traits of OPV compared to its inorganic and hybrid counterparts (mainly represented by silicon- and perovskite-based PV) are their low cost, environmentally-friendly processing and sustainability.^5–7^ These are, however, still compromised by the use of hazardous halogenated solvents to deposit the photoactive layer (PAL) and the large synthetic complexity^8^ (SC) of its raw materials, namely the donor (p-type) and acceptor (n-type) semiconductors. The SC, as introduced by Po *et al.* in 2015, aims at quantifying the experimental effort required in the synthesis of organic semiconducting materials for OPV,^8^ so that lower SC materials have larger industrial impact and potential upscalable synthesis.^9^ Therefore, the identification of non-halogenated and low SC PAL ink formulations offering high PCE is key to enable OPV upscaling in mass-printing methods such as roll-to-roll (R2R), thus fulfilling the desired functionalities offered by the OPV technology.

**Fig. 1:**
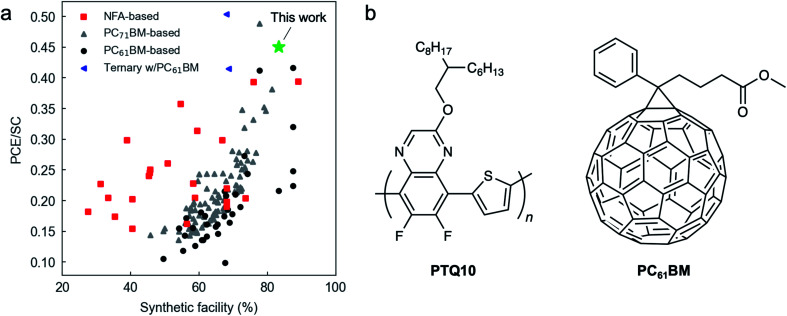
(a) Power conversion efficiency (PCE, %) to synthetic complexity (SC, %, as defined by Po *et al.*)^8^ ratio for reported binary and ternary BHJ devices as a function of the synthetic facility (SF, %) of the active layer components. The SF is computed as 100 – SC. (b) Chemical structures of the donor (PTQ10) and acceptor (PC_61_BM) materials used in this work.

Regioregular poly(3-hexylthiophene-2,5-diyl) (P3HT) represents the archetypal p-type conjugated homopolymer in OPV with the lowest SC up to date (7.6%, see Table S1†),^8,10^ which already opened the possibility to be synthetized in gram-sized, continuous-flow batches.^11^ Accordingly, P3HT has been widely studied in potential upscalable combinations with fullerene^12^ and non-fullerene acceptors (NFAs).^13–16^ In combination with NFAs, P3HT blends have reached close to 9.5% PCE (under 1 sun) in binary bulk heterojunctions (BHJs)^16,17^ by exploiting the chemical tunability of their structures and the positioning of their frontier energy levels (highest occupied molecular orbital, HOMO; and lowest unoccupied molecular orbital, LUMO). However, the SC of the best performing NFAs is at least doubling that of the workhorse fullerene-based acceptors such as [6,6]-phenyl-C_61_-butyric acid methyl ester (PC_61_BM) and [6,6]-phenyl-C_71_-butyric acid methyl ester (PC_71_BM), which hinders their adoption onto industrial scenarios (Fig. 1a and Table S1†). Among fullerenes, PC_61_BM is the preferable choice due to its lower cost per gram and good solubility, yet the PCE in P3HT:PC_61_BM BHJs is limited to 5.2% under 1 sun,^18^ and to 12.8% under LED illumination.^19^

The introduction of poly[(thiophene)-*alt*-(6,7-difluoro-2-(2-hexyldecyloxy)quinoxaline)] (PTQ10, Fig. 1b) as a thiophene-based p-type copolymer^20^ quickly turned focus onto such materials as a natural upgrade and replacement of P3HT. PTQ10 offers a SC comparable to that of fullerenes,^21^ and less than 10 percentage units higher than that of P3HT^17^ (15.9% and 7.6% for PTQ10 and P3HT, respectively, as detailed in Table S1†); remarkably, PTQ10 shows PCEs in excess of 16% (under 1 sun) in combination with benchmark NFAs such as Y6 (BTP-4F)^17^ and some of its isomeric variants (17.7% PCE).^22^ For indoor applications, PTQ10:NFA-based devices demonstrate open-circuit voltages (*V*_oc_s) beyond 1 V,^23^ and decent PCEs of 15.7%.^24^ Conversely, the PCE of PTQ10 in combination with fullerenes (PC_61_BM) appears limited to 3.6% only (under 1 sun),^21^ which might be a result of non-optimized OPV device architectures. To the best of our knowledge, examples targeting indoor OPV applications of a PTQ10:fullerene-based PAL are still absent.

Amongst the different device parameters, the photovoltaic performance can be significantly boosted by incorporating into the solar cell contacts charge selective interlayers, namely the electron and hole transport layers (ETL and HTL, respectively). The choice of ETL and HTL materials is of striking importance in maximizing the PCE^25^ of inverted device architectures (which are those with the largest industrial interest)^26^ as (i) they must guarantee barrierless charge transfer of electrons and holes from the BHJ to the contact (ohmic junction), otherwise, the *V*_oc_ will be reduced; and (ii) they must form a compatible and stable interface with the BHJ to enhance the short-circuit current density (*J*_sc_), fill factor (FF) and device lifetime.^27^

On top on the abovementioned requirements in terms of PCE and SC, PAL and interlayer combinations suitable for upscaling should also be able to retain performance in thick layers^28^ (>200 nm and 50–100 nm for the PAL and interlayer, respectively) as well as through inherent thickness fluctuations that occur during industrial manufacture (*i.e.*, thickness sensitivity),^29^ for instance in slot-die coating R2R setups. Ink formulations based on non-halogenated solvents are also desirable to facilitate device manipulation in air and avoid collateral human health and environmental effects. Finally, the combination of charge selective interlayers (ETL and HTL) and PAL should also demonstrate compatibility with R2R upscaling, namely in terms of air processability and device stability.

In this work we present a non-halogenated PAL ink formulation based on PTQ10:PC_61_BM that offers PCEs as high as 7.5% under simulated AM1.5G irradiance conditions, and 19.9% under indoor LED illumination. The record performance in such versatile and low SC PAL arises from (i) an optimized co-solvent ink formulation based on *o*-xylene and diphenyl ether; and (ii) proper compatibility matching between the BHJ and its underlying layer, *i.e.*, the ETL in inverted device architectures. Zn-based ETL formulations are found to work best with the here optimized PAL ink under sun conditions. The record PCE is achieved using a methanol-based polyethyleneimine-Zn (PEI-Zn) chelated ETL ink^30,31^ that provides FFs as high as 74%, which also offers a strong robustness against ETL and PAL thickness fluctuations over a wide interval (extending up to 150 nm for the ETL and 200–400 nm for the PAL). Under indoor illumination conditions, SnO_2_ shows superior performance while avoiding UV-induced photodoping for proper functioning. Based on the recombination analysis of devices processed with up to 4 different ETLs, we show that the choice of interlayer largely affects the magnitude of mono- and bimolecular recombination mechanisms in the PAL. As a result, PTQ10:PC_61_BM and its corresponding PEI-Zn and SnO_2_ ETLs are currently positioned as one of the most versatile and industrially relevant binary OPV systems given their high performance and non-halogenated formulation with intrinsically low SC and cost. In fact, our optimized PTQ10:PC_61_BM system constitutes a new PCE record under 1 sun below the 20% SC threshold (or, alternatively, above the 80% synthetic facility (SF) threshold, see Fig. S1†). Furthermore, by introducing the PCE/SC ratio as a metric proportional to the industrial figure of merit (i-FoM) in solution-processed OPV,^32^ our results highlight as a new PCE/SC record among PC_61_BM-based, binary organic solar cells (Fig. 1a). Finally, we prototype laminated devices entirely in air that are based on the SnO_2_ and PTQ10:PC_61_BM material system combination for indoor photovoltaic applications, in a way congruent with R2R manufacturing. We obtain PCEs exceeding 10% under 500 lux illumination conditions, while thermal and photostability data suggest that more than 80% of the initial performance is retained after 400 and 350 hours of thermal and light stress, respectively. These figures further support the compatibility of the PTQ10:PC_61_BM blend for R2R upscaling.

## Results and discussion

### Optimization of the PAL ink co-solvents and thickness

We start by optimizing the PAL ink formulation using our group-standard (and previously optimized)^33^ ETL formulation: a commercial ZnO nanoparticle dispersion provided by Avantama (N-10, based on 2-propanol). To limit the extension of the parameter space, the donor-to-acceptor ratio is kept fixed at 1 : 1.5 (w/w) throughout the experiments, targeting a total solid content of 30–50 g L^−1^. *o*-Xylene is selected as primary solvent of the PAL ink due to its better compatibility with upscaling, as being a non-halogenated solvent less hazardous for human health and the environment. The PAL ink formulation is thus screened in terms of co-solvent fractions, which are usually referred to as additives. These have been shown to have a dramatic effect on the BHJ film morphology and thus on the final device performance.^34–37^ 1,8-Diiodooctane (DIO) is one of the most frequent additives used in fullerene-rich BHJs to boost the device performance at the expense of a compromised long-term stability,^34,35^ as DIO is difficult to be completely removed from the PAL.^38^ Alternative, more volatile and less hazardous additives such as diphenyl ether (DPE) are now gaining attention^36,37^ as they offer similar performance enhancements with respect to DIO while guaranteeing an easier removal of additive in the dried PAL.

We thus add DIO or DPE in varying volume fractions to the pristine *o*-xylene carrier solvent and compare the observed PCEs as a function of the PAL thickness. Starting from the neat PAL ink formulation (100 : 0, v/v, *o*-xylene : additive), DIO-rich inks are screened at 1 vol% (99 : 1) and 5 vol% (95 : 5) steps, while DPE-rich inks are explored in larger steps of 5 vol% (95 : 5), 10 vol% (90 : 10) and 15 vol% (85 : 15). To optimize the raw material consumption and speed up the screening process, we deposit the corresponding PALs as thickness gradients *via* accelerated blade coating.^13,33,39^ This approach screens 12 different parametric combinations with two replicates each (thus creating 24 devices or pixels in a single large aspect-ratio, see Fig. S2†), with a distribution of the final PCE resulting from the lateral PAL thickness variation along the substrate (see Fig. S3 and S4†). The PCE of the champion devices using N-10 as ETL are summarized in Table 1 (a total of 240 devices were characterized). Despite showing a high *V*_oc_ of 1.00–1.01 V, the pristine ink (100 : 0) shows very poor *J*_sc_ (<2 mA cm^−2^), FF (<50%) and PCE (<1%). Clearly, co-solvents have a positive effect on device performance. The addition of DIO monotonically increases *J*_sc_ and FF at the expense of slightly reduced *V*_oc_; when using N-10 as ETL, the best performance is achieved adding 5 vol% DIO (*V*_oc_ = 0.95 V; *J*_sc_ = 9.59 mA cm^−2^; FF = 62%; PCE = 5.67%). In the case of DPE, the relationship is no longer monotonic, but the performance increases abruptly when 15 vol% DPE is added as co-solvent. For the champion device, all figures-of-merit (*V*_oc_ = 0.96 V; FF = 70%; PCE = 6.35%) but *J*_sc_ (9.37 mA cm^−2^) overcome the values attained using 5 vol% DIO. In overall, we observe that the champion PCEs in these two optimized co-solvent cases represent up to an 8-fold improvement with respect to the initial performances (*i.e.*, the neat *o*-xylene solvent system), thus highlighting the relevance of the precise PAL ink formulation.

**Table 1:** Photovoltaic figures-of-merit of the champion PTQ10:PC_61_BM devices obtained as a function of the PAL ink co-solvent system (either DIO or DPE added to the neat *o*-xylene) and choice of ETL (reference N-10 or N-31). A total of 312 devices were characterized in these screening steps

ETL formulation	PAL ink co-solvent	*V*_oc_ (V)	*J*_sc_ (mA cm^−2^)	FF	PCE (%)
N-10 (ZnO)	Neat	1.01	1.52	0.49	0.76
	1 vol% DIO	0.98	2.58	0.63	1.58
	5 vol% DIO	0.95	9.59	0.62	5.67
N-31 (SnO_2_)	5 vol% DIO	0.96	10.86	0.68	7.09
N-10 (ZnO)	Neat	1.00	1.71	0.47	0.80
	5 vol% DPE	0.92	0.84	0.32	0.25
	10 vol% DPE	0.70	0.92	0.36	0.23
	15 vol% DPE	0.96	9.37	0.70	6.35
N-31 (SnO_2_)	15 vol% DPE	0.94	8.79	0.71	5.90

We then demonstrate that the conclusions drawn in terms of PAL ink formulation can be translated with similar (and even improved) performance when a different ETL is introduced in the inverted device architecture. In this case, we substitute the ZnO N-10 dispersion by a commercial SnO_2_ ETL formulation (N-31 by Avantama, based on a mixture of butanols) while testing the top-performing PAL ink formulations previously found (additional 72 devices were characterized). As shown in Table 1, the optimized 5 vol% DIO counterpart in combination with N-31 shows an improved PCE of 7.09%, which is attributed to the enhanced *V*_oc_, *J*_sc_ and FF reaching 0.96 V, 10.86 mA cm^−2^ and 68%, respectively. Conversely, the 15 vol% DPE PAL ink processed atop N-31 shows lower *V*_oc_ (0.94 V) and *J*_sc_ (8.79 mA cm^−2^) than the N-10 counterpart, while the FF increases up to 71%; as a result, the PCE drops to 5.90%. Hence, SnO_2_ improves the FF but not necessarily the PCE, as *V*_oc_ and *J*_sc_ might drop depending on the PAL ink formulation used. These findings suggest that the compatibility of the PAL components and the adjacent ETL is yet another important variable to consider in the device optimization. We hypothesize that some of the underlying phenomena that could be affected by the choice of ETL in an inverted device are the wettability of the PAL ink and its subsequent vertical phase segregation and morphology once dried as BHJ.^40^

**Fig. 2:**
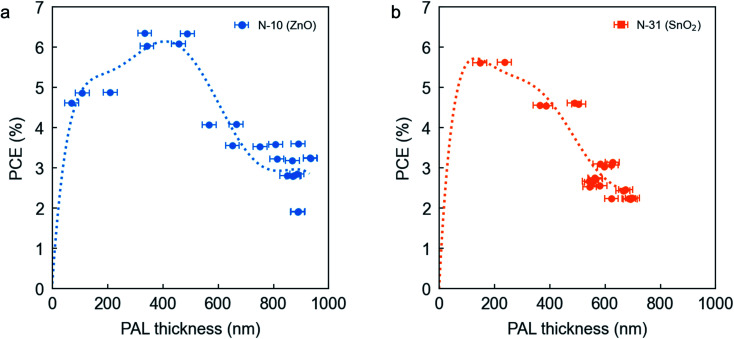
PCE as a function of PAL thickness for devices processed using N-10 (a) or N-31 (b) as ETL and 15 vol% DPE as co-solvent. Dotted lines are polynomial fits to the data serving as guide-to-the-eye.

For the rest of the manuscript, we will focus on the PAL ink formulation that we believe shows the largest industrial interest for its environmental friendliness and uncompromised performance (*i.e.*, 15 vol% DPE). Further characterization of the PAL thicknesses obtained in the top performing DPE-based devices reveals that the PTQ10:PC_61_BM binary finds its absolute PCE maximum at *ca.* 400 nm when N-10 is employed as ETL (Fig. 2a). The occurrence of such maximum for thick PALs (>200 nm)^28^ requires a narrow distribution of tail states to avoid charge accumulation and internal electric field screening, which otherwise lead to *J*_sc_ losses.^41^ High FFs for thick PALs (*ca.* 70%, see Fig. S6c†) also indicate limited transport and recombination losses,^42^ so that higher-order interference maxima for *J*_sc_ are experimentally plausible. Notably, Fig. S6b† shows a rather flat *J*_sc_ extending from 300 to 700 nm of PAL thickness, in close agreement with our optical simulations assuming 65% internal quantum efficiency (IQE). Note that for most OPV PALs and as the charge transport losses become increasingly important with PAL thickness (leading to a drop in FF),^43,44^ the oscillation of *J*_sc_ derived from optical interference is limited to the first-order peak (70–100 nm) and only the saturated reverse bias current follows the modelled higher-order interference pattern.^45^ However, the optimized morphology and good transport properties of the PTQ10:PC_61_BM blend system in combination with N-10 as ETL allows observation of higher-order interference patterns even for *J*_sc_. Conversely, when N-31 is the option of choice the maximum PCE shifts downwards to *ca.* 200 nm (Fig. 2b) due to serious limitations on the FF at large PAL thicknesses (<60%, Fig. S7†). In general, PAL thicknesses beyond these threshold values (400 and 200 nm when using N-10 or N-31, respectively) give rise to an steady decrease in FF and *J*_sc_ (Fig. S6 and S7†) as expected from the limited charge transport capabilities of organic semiconductors.^42^ Note that in this binary ETL case study (N-10 *vs.* N-31), the distinct optical constants of ZnO and SnO_2_ might be largely affecting the distribution of the electromagnetic field in the PAL; this might influence *per se* the location of the corresponding *J*_sc_ maxima as a function of PAL thickness. In order to enable the transfer matrices modelling of the upper *J*_sc_ limit in N-10-based devices (Fig. S6b†), we have measured the absorbance spectrum and modelled the complex refractive index through ellipsometry of a blade coated PTQ10:PC_61_BM film with 15 vol% DPE (Fig. S5†). Still, the PTQ10:PC_61_BM can be classified as a thick-active-layer binary blend as the occurrence of performance maxima beyond 100 nm in PAL thickness is rather unique of selected binary systems only,^28,40,41,46^ and of critical importance for OPV upscaling.^29^

### ETL screening: formulations and thickness

With the observed influence of the ETL choice on device performance, we extend the screening study to some commercial ETL formulations as well as others reported in literature. The criteria followed in the selection of ETLs in this work is based on their commercial availability and frequent use among other researchers in the OPV field. As an alternative formulation to the workhorse ZnO (N-10), we consider a commercial ink provided by infinityPV (based on 2-propanol). We further select Al:ZnO (N-21X-Flex by Avantama, based on a mixture of butanols) due to its compatibility with R2R and flexible substrates. Previously, we also consider N-31 (SnO_2_) as an alternative oxide for indoor applications; (further details about these commercial formulations are found in Table S2†). We further reformulate a recently reported PEI-Zn chelated ink^30,31^ in which we substitute the original solvent 2-methoxyethanol by less hazardous methanol (further details are provided in the Methods section). The PEI-Zn chelating strategy shows potential for mitigating the photocatalytic activity of Zn cations, thus extending the device lifetime. Note that non-halogenated solvents are neither employed in any of the ETL formulations herein considered. In this ETL screening study, we exploit thickness gradients prepared by decelerated blade coating to evaluate the effect that ETL thickness has in device performance in a high-throughput fashion (Fig. S8a†). However, we also observe the formation of an unintended PAL thickness gradient attributed to ink depletion during blade coating (Fig. S8b†), which increases correlation between PAL and ETL thickness values in our study (Fig. S12 and S13†).

**Fig. 3:**
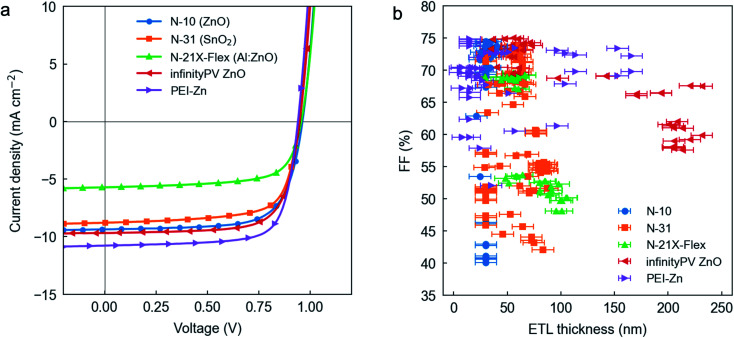
(a) *J*–*V* curves of the champion solar cells obtained for each ETL under simulated AM1.5G irradiance (1 sun, 1000 W m^−2^). (b) FF as a function of ETL thickness for the different interlayers tested in this work.

Fig. 3a shows the *J*–*V* curves of the champion solar cells obtained under simulated 1 sun AM1.5G irradiance for each of the tested ETLs. Their corresponding figures-of-merit are shown in Table 2, in which we acknowledge that *J*_sc_ is the parameter that shows the largest variability in relative terms. According to their record PCE values, the Al:ZnO formulation (N-21X-Flex by Avantama) lags behind in terms of performance with a champion PCE of only 3.76%, resulting from a hampered *J*_sc_ of 5.72 mA cm^−2^ and a FF right below 70% (69%). All the remaining ETL formulations show FFs equal to or surpassing 70% together with significantly improved *J*_sc_ values. We observe that in combination with the 15 vol% DPE PAL ink formulation, SnO_2_ slightly underperforms with respect to Zn-based ETLs (N-10, infinityPV ZnO and PEI-Zn). In particular, the infinityPV ZnO formulation achieves improved *V*_oc_, *J*_sc_ and FF with respect to SnO_2_ (0.95 and 0.94 V; 9.68 and 8.79 mA cm^−2^; 72 and 71% FF, respectively); as a result, the champion PCE raises up to 6.62% (from the 5.90% achieved using SnO_2_). Nevertheless, our record PCE for the PTQ10:PC_61_BM blend is attained using the methanol-based PEI-Zn chelated strategy as ETL. The reader should note that for the different ETLs, we used the annealing protocols that had been previously optimized in the group (see Methods section) excepting PEI-Zn, whose optimization is first reported here as constituting a novel ETL formulation. Accordingly, we first optimize the annealing temperature of such interlayer by generating a linear temperature gradient distributed along the 24 pixels of a single substrate, which includes a homogeneous PEI-Zn thickness. The results shown in Fig. S9† suggest that an annealing temperature of *ca.* 130 °C maximizes the PCE within the temperature range explored (104–148 °C), thus constituting our option of choice in the remaining parts of the present study. A representative *J*–*V* curve for PEI-Zn devices annealed at 130 °C together with the external quantum efficiency (EQE) and the integrated *J*_sc_ are provided in Fig. S10.† As a result, for the optimized PEI-Zn device we observe that the increase in *J*_sc_ (10.78 mA cm^−2^) and FF (74%) counterbalances the slight decrease in *V*_oc_ (0.94 V) to reach a maximum PCE of 7.51%. Such record PCE value lies among the highest ever reported for a donor:acceptor blend based on PC_61_BM (see Fig. S1,† only exceeded by PffBT4T-2OD:PC_61_BM with 9.2% PCE yet showing a SF below 80%).^47^ In terms of PCE/SC ratio (in close analogy to the i-FoM),^32^ our PTQ10:PC_61_BM optimized system also positions as one of the most industrially-relevant OPV blends (PCE/SC = 0.45, which corresponds to a new record value among PC_61_BM binary blends and also above the 80% SF threshold, see Fig. 1a). Finally, the straightforward and low cost preparation of the PEI-Zn interlayer ink (requiring three raw components only, namely zinc acetate dihydrate, PEI and methanol) adds further value to our findings in potential life cycle analysis (LCA) of OPV devices,^7^ which render critical to maximize the sustainability of the OPV technology.

**Table 2:** Photovoltaic figures-of-merit, series resistance (*R*_s_) and shunt resistance (*R*_sh_) of the champion devices processed using different ETL formulations with the pre-optimized PAL ink containing 15 vol% DPE and *o*-xylene as carrier solvent. Note that both resistances were estimated from the *J*–*V* curves under 1 sun illumination conditions

ETL formulation	*V*_oc_ (V)	*J*_sc_ (mA cm^−2^)	FF	PCE (%)	*R*_s_ (Ω cm^−2^)	*R*_sh_ (kΩ cm^−2^)
N-10 (ZnO)	0.97	9.37	0.70	6.35	2.09	3.26
N-31 (SnO_2_)	0.94	8.79	0.71	5.90	1.86	3.18
N-21X-Flex (Al:ZnO)	0.96	5.72	0.69	3.76	2.32	2.34
infinityPV ZnO	0.95	9.68	0.72	6.62	1.98	4.65
PEI-Zn	0.94	10.78	0.74	7.51	2.33	2.42

To assess the effect of ETL thickness fluctuations on device performance, we turn focus onto the FF as being the most sensitive device parameter to charge transport, charge selectivity, and charge recombination processes alone. Fig. 3b includes FF *vs.* ETL thickness data for >300 devices, whereas the corresponding *J*_sc_ and PCE values are shown in Fig. S11a and b,† respectively. Therein, the reader should note that we include datapoints with homogeneous ETL thickness (*ca.* 30 nm for N-10 and N-31; *ca.* 15 nm for PEI-Zn) corresponding to the devices shown in Fig. 2, which are prepared in the form of PAL thickness gradients only. We generally observe that blade coating deceleration leads to ETL thickness gradients extending from 100–150 nm to 20 nm (Fig. S8a†), which results from the similar rheology and solid content of the ETL inks (Table S2,† excepting PEI-Zn). For all the ETLs tested in this work, the corresponding FFs generally reach their highest values in a thickness regime centred around 50 nm, with little to no effect upon thickness fluctuations of ±25 nm. Thus, as a rule of thumb, an ETL thickness of *ca.* 50 nm leads to a close-to-optimum FF (also enabling maximum *J*_sc_ and PCE, see Fig. S11†) for all tested ETLs. N-31 and N-21X-Flex are found to slightly decrease their performance when exceeding 75 nm in thickness in combination with moderately thick PALs (200–300 nm), showing FFs below 60%; see also Fig. S12 and S13† for correlation plots between ETL thickness, PAL thickness and performance (FF and *J*_sc_, respectively). Beyond 100 nm, only PEI-Zn is found to maintain FFs above 70%, thus gaining special attention for upscalable approaches; conversely, formulations such as the infinityPV ZnO show an evident decrease in performance when thicker ETLs are employed (FFs <70%) even in combination with thin PALs (<100 nm, see Fig. S12e†). However, the infinityPV ZnO ETL shows an impressive and very robust PCE distributed over a wide range of ETL thickness values extending from 25 to 75 nm (with an average PCE of (6.5 ± 0.1)%, Fig. S11b†). On the other hand, the PEI-Zn ETL is found to maintain a rather flat PCE extending from 10 to 150 nm in thickness (Fig. S11b†) yet in this particular ETL thickness screening experiment the PCE remained limited to (4.1 ± 0.2)%. Note that the observed dispersion throughout the *y*-axis in Fig. 3b (and Fig. S11a, b†) is attributed to both the intrinsic reproducibility of the device fabrication and to the unintended PAL thickness variations (200–350 nm) arising from PAL ink depletion during blade coating (Fig. S8b†). This is observed to yield a positive correlation between ETL and PAL thicknesses for the N-31 and N-21X-Flex cases (Fig. S12 and S13†).

### Recombination analysis of the ETLs

**Fig. 4:**
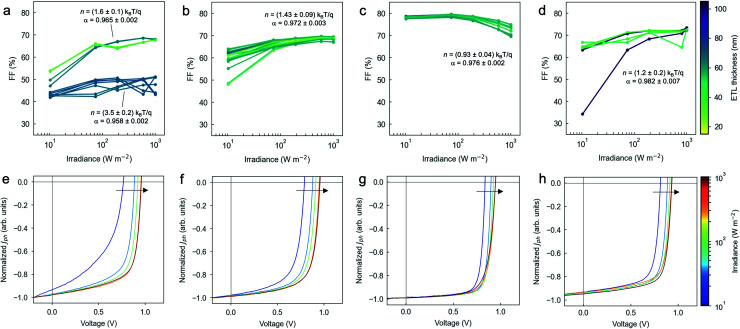
FF and representative normalized *J*–*V* curves as a function of irradiance (*Φ*) for four different ETLs: (a, e) N-31; (b, f) N-21X-Flex; (c, g) infinityPV ZnO; and (d, h) PEI-Zn. The corresponding light ideality factors (*n*) observed for *V*_oc_ and the fitting constant *α* for *J*_sc_ are also included for each subset of datapoints. For the upper plots, the line colours represent the ETL thickness. The arrows on the lower plots indicate the overall trend of the *J*–*V* curves as irradiance increases (also illustrated with the colour of the curves). *J*–*V* curve normalization is performed by subtracting the dark *J*–*V* curve (*J*_dark_) to the illuminated *J*–*V* curve (*J*_light_) as *J*_ph_ = *J*_light_ − *J*_dark_, and normalizing the resulting *J*–*V* curve (*J*_ph_) by its value at large reverse bias (*J*_ph,sat_, measured at −0.5 V in our case): *J*_ph,normalized_ = *J*_ph_/*J*_ph,sat_.^51^

We further investigate the corresponding device physics by performing a light intensity-dependent recombination analysis of our catalogue of ETLs, also including variations of the ETL thickness. The light ideality factor (*n*) is obtained by fitting the measured *V*_oc_ as a function of irradiance (*Φ*) according to the following equation:^48^
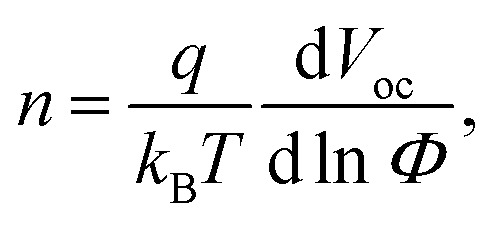
where *q* is the elementary charge, *k*_B_ is the Boltzmann's constant and *T* is the absolute temperature. Ideality factors close to unity indicate that bimolecular recombination is dominant in open-circuit conditions, with very limited monomolecular and trap-assisted recombination.^49^ Similarly, by fitting *J*_sc_ to a power law given by *J*_sc_ ∝ *Φ*^*α*^ we detect the occurrence of bimolecular recombination and space-charge in the device under short-circuit conditions.^50^ In particular, *α* values approaching unity indicate the absence of bimolecular recombination, so that monomolecular recombination is the major recombination process limiting the performance.^51^Fig. 4 shows the FF *vs.* light intensity-dependence observed in more than 200 devices with different ETLs (N-31, N-21X-Flex, infinityPV ZnO and PEI-Zn) and thicknesses (ranging from 15 to 105 nm and quantified using a colour scale); the remaining photovoltaic parameters (*V*_oc_, *J*_sc_ and PCE) are shown in Fig. S14 and S15.† The average ideality factors together with the corresponding *α* coefficients required to fit *V*_oc_ and *J*_sc_ in semi-log and log–log plots as a function of irradiance (Fig. S14 and S15†) are also detailed in Fig. 4. Note that during the acquisition of all *J*–*V* curves, we verified that the measurements were properly stabilised during a period of at least 5 minutes.

N-31 (Fig. 4a, e) and N-21X-Flex (Fig. 4b, f) interlayers show similar FF trends, with *α* values reading 0.965 ± 0.002 and 0.972 ± 0.003, respectively; and ideality factors ranging from (1.6 ± 0.1) *k*_B_*T*/*q* to (1.43 ± 0.09) *k*_B_*T*/*q*, respectively. Interestingly, we find the FF to increase asymptotically while being positively correlated with the irradiance (also in PEI-Zn, see Fig. 4d), a trend that is no longer reproduced when employing infinityPV ZnO as ETL (Fig. 4c). Ideality factors for infinityPV ZnO ((0.93 ± 0.04) *k*_B_*T*/*q*, Fig. 4c) and PEI-Zn ((1.2 ± 0.2) *k*_B_*T*/*q*, Fig. 4d) interlayers are closer to unity than the remaining candidates, thus indicating the absence of a strong monomolecular recombination in open-circuit conditions; this is in good agreement with the trends observed in their corresponding normalized *J*–*V* curves (Fig. 4g and h).^51^ The statistical occurrence of ideality factors right below unity (as per infinityPV ZnO case) suggests potential degradation in the device (especially affecting the PAL) under 1 sun illumination conditions, which drives a levelling off in the *V*_oc_ trend in the corresponding semi-log plot (Fig. S14c†). We observe that *α* values can be as high as 0.976 ± 0.002 and 0.982 ± 0.007 for the infinityPV ZnO and PEI-Zn interlayers, respectively, which enable close to 80% FF at low irradiances (*ca.* 10–100 W m^−2^) for the infinityPV ZnO case; and average FFs above 60% regardless the input irradiance in both cases. Nevertheless, in the infinityPV ZnO scenario the FF is observed to drop significantly when thick ETLs (>100 nm) are incorporated into the device stack (Fig. 3b), which we attribute to a decrease in *α* that adds bimolecular recombination losses at short-circuit conditions. In this regard, only the PEI-Zn interlayer is found to maintain the FF within competitive figures (>70%) even at such large thicknesses, thus constituting an ideal candidate for OPV upscaling.

We also quantify the exciton dissociation efficiency (*P*_diss_) and charge collection efficiency (*P*_coll_) for some of the most representative ETLs and best performing devices. Based on the analysis of their corresponding photocurrent *versus* effective voltage curves (Fig. S16†),^49,52^ we find that *P*_diss_ exceeds 98% in all three ETLs analysed (98.98%, 99.76% and 99.84% for N-21X-Flex, infinityPV ZnO and PEI-Zn, respectively). Similarly, *P*_coll_ increases from 74.42% for N-21X-Flex to 85.74% for PEI-Zn while reaching 87.56% in the champion infinityPV ZnO device.

Therefore, the outcomes of the recombination analysis suggest that the asymptotic behaviour of the FF *vs. Φ* curves is due to photoinduced doping of the ETLs, which might be acting to improve its charge extraction properties such as charge mobility and charge density as irradiance increases. Our results also indicate that the infinityPV ZnO layer might be excessively resistive, thus Joule's effect losses increase with the current flowing through the device (*i.e.*, *Φ*) while constraining the performance (FF). Finally, the lower performance of the N-21X-Flex (Al:ZnO) ETL could be attributed to a strongly different conductivity with respect to the neat ZnO-based counterparts, as expected from the incorporation of Al in the bulk of the interlayer. Regrettably, this is found to negatively affect FF and *J*_sc_ when employing this ETL (Table 2). However, further experiments out of the scope of this manuscript are required to reach full understanding of these observations.

### Indoor photovoltaic performance

**Fig. 5:**
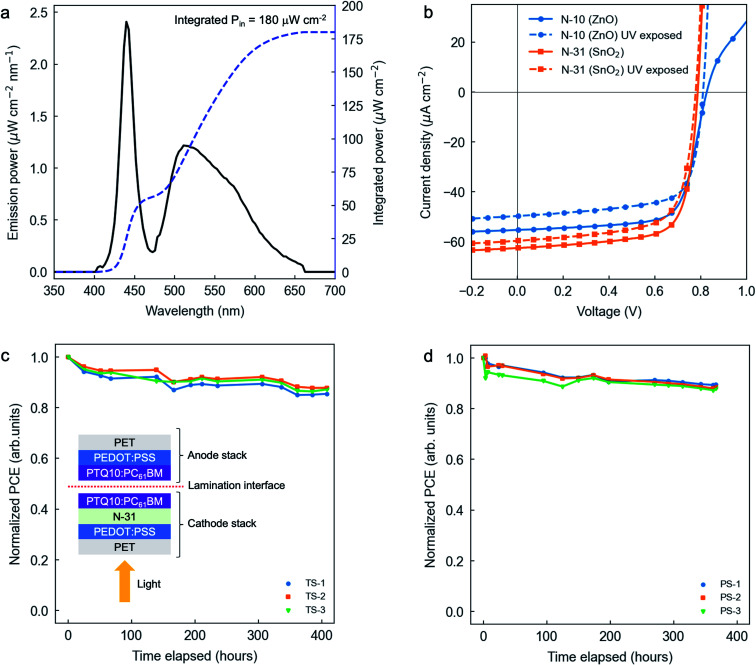
(a) Indoor LED emission power spectrum (solid black line) and integrated input power (*P*_in_, blue dashed line). (b) Representative *J*–*V* curves for N-10 (blue circles, as cast (solid line) and UV exposed (dashed line)) and N-31 (orange squares, as cast (solid line) and UV exposed (dashed line)) as ETLs under indoor irradiance conditions. (c) Thermal stability study of three different laminated devices annealed at 70 °C in a nitrogen-filled glovebox and in the dark. The inset shows a cross-sectional sketch of those same laminated devices. The cathode and anode stacks are processed independently and brought together at the lamination interface. The device is then illuminated from the cathode side. (d) Photostability study under 500 lux illumination conditions for three different laminated devices while in a nitrogen-filled glovebox. The irradiance spectrum of the light source used on the stability studies is shown in Fig. S19.†

The high band gap of the PTQ10:PC_61_BM blend (Fig. S5†) and the excellent performance observed under low AM1.5G irradiance conditions (particularly the FF, indicating high *R*_sh_) motivate using such OPV system for indoor photovoltaic purposes. Accordingly, we assess its photovoltaic performance under typical indoor LED illumination conditions (Fig. 5a) in combination with two archetypal ETLs, namely N-10 (ZnO) and N-31 (SnO_2_). The corresponding figures-of-merit of champion devices are detailed in Table 3, while their *J*–*V* curves are depicted in Fig. 5b.

**Table 3:** Indoor photovoltaic figures-of-merit, *R*_s_ and *R*_sh_ of the champion devices processed using N-10 and N-31 as ETL. The input power (*P*_in_) reads 180 μW cm^−2^. Note that *R*_s_ and *R*_sh_ were extracted from the *J*–*V* curves under illumination conditions

ETL formulation	Conditions	*V*_oc_ (V)	*J*_sc_ (μA cm^−2^)	FF	PCE (%)	*R*_s_ (Ω cm^−2^)	*R*_sh_ (kΩ cm^−2^)
N-10	As cast	0.83	55.3	0.71	18.1	8745	277.2
	UV exposed	0.81	49.7	0.71	15.9	7.90	183.2
N-31	As cast	0.79	62.5	0.73	19.9	7.68	196.1
	UV exposed	0.78	59.7	0.70	18.0	7.40	167.8

We first observe that the as cast ZnO formulation imposes a serious limitation on the series resistance (*R*_s_) of the device as suggested by the narrower slope of the *J*–*V* curve in forward bias (Fig. 5b and Table 3). However, given our illumination conditions (*P*_in_ = 180 μW cm^−2^), the higher *R*_s_ is not enough to significantly limit the photovoltaic performance, which reaches a decent PCE of 18.1% and a FF of 71%. By remeasuring the same device after UV exposure (also referred to as light soaking,^53^ which in our case corresponds to 1 sun irradiance conditions for *ca.* 5–6 minutes), we observe an acute decrease in *R*_s_, thus suggesting that photodoping is taking place in the ZnO interlayer.^54,55^ We acknowledge that UV exposure is negatively affecting the device performance (as these are unencapsulated devices), which lowers *V*_oc_ (from 0.83 to 0.81 V), *J*_sc_ (from 55.3 to 49.7 μA cm^−2^) and PCE (from 18.1 to 15.9%) figures with respect to pristine devices. Photodoping of ZnO-based interlayers is further evidenced when employing PEI-Zn as ETL; in this case, we observe the occurrence of an S-shaped *J*–*V* curve under LED illumination that is completely vanished under 1 sun (Fig. S17†). Therefore, ZnO-based ETLs are generally not suitable for strictly indoor OPV applications.

On the other hand, SnO_2_ as found in its commercial formulation N-31 does not show a compromised *R*_s_ in as cast films, which suggests that UV exposure is unnecessary to unlock the conductivity of the ETL. This is in good agreement with the nearly absent electron de-trapping from sub-gap states reported for SnO_2_ films.^53^ Due to its higher work function (Table S2†), N-31 devices show limited *V*_oc_ (0.79 V) compared with N-10 yet they offer improved *J*_sc_ (62.5 μA cm^−2^) and FF (73%), which raise PCE very close to 20% (19.9% in as cast devices). UV exposure is also found to negatively affect the device performance by dropping all figures-of-merit yet without significant effects on *R*_s_. Therefore, ETL formulations based on SnO_2_ rather than ZnO offer a superior performance for indoor photovoltaic applications and do not require a UV-induced photoactivation step that might be followed by potential device degradation. These features are of special interest and acknowledged for upscaling of indoor OPV devices.

### Prototyping and stability of upscalable laminated devices for indoor photovoltaics

In this last section, we demonstrate the potential scalability of organic solar cells based on the ETL (N-31) and PAL (PTQ10:PC_61_BM) previously optimized for indoor photovoltaic applications. These devices are manufactured *via* lamination,^56^ entirely in air, on flexible polyethylene terephthalate (PET) substrates, with an inverted architecture and avoiding the use of either indium tin oxide (ITO) or evaporated metal contacts. These are all procedures congruent with R2R requirements. As per the lamination technique, two device sub-stacks are processed independently and then brought together by applying pressure and temperature to the stacks while placed in between two counter-rotating rollers; see inset of Fig. 5c for a cross-sectional sketch of a laminated device. The cathode side stack includes a slot-die coated poly(3,4-ethylenedioxythiophene):polystyrene sulfonate (PEDOT:PSS) layer as electrode, followed by a blade coated N-31 layer as ETL and blade coated PTQ10:PC_61_BM as PAL. Conversely, the anode side stack is composed by a slot-die coated layer of PEDOT:PSS (which acts as electrode and HTL simultaneously) and a blade coated PAL atop. The laminated devices are semi-transparent (with an average visible transmittance of 3.5%, see Fig. S18†) while showing record FFs of 71.5% and a champion PCE of 10.4% under 500 lux illumination conditions (equivalent to 141.3 μW cm^−2^, see Fig. S19†). The photovoltaic figures-of-merit of up to 6 different laminated devices, whose active area is defined using two different apertures of either 0.25 or 0.07 cm^2^, are collected in Table S3.† Their average *V*_oc_, *J*_sc_, FF and PCE read (0.72 ± 0.03) V; (28 ± 3) μA cm^−2^; (70 ± 1)%; and (9.8 ± 0.7)%, respectively.

To further support the compatibility of the here presented ETL and PAL combination with upscalable R2R manufacture, we assessed the thermal and photostability of such air-processed laminated devices. The thermal stability study is performed in an oven at 70 °C in a nitrogen-filled glovebox and in the dark. Our results (Fig. 5c) indicate that the laminated devices require more than 400 hours of thermal stress to drop their PCE to 80% of its initial value (thermal-T_80_). As per the *J*–*V* curve characteristics, thermal stress is observed to affect mostly *V*_oc_ and FF, with more limited effect on *J*_sc_ (Fig. S20a†). As far as the photostability study is concerned, the laminated devices are exposed to continuous 500 lux illumination conditions in a nitrogen-filled glovebox. Fig. 5d shows that air-processed laminated devices have an associated photo-T_80_ value that exceeds 350 hours. According to their representative *J*–*V* curves, the main loss in performance is ascribed to a decreased *J*_sc_ while showing an excellent retainment of *V*_oc_ and FF (Fig. S20b†). As a result, the proposed combination of N-31 as ETL and PTQ10:PC_61_BM as PAL demonstrates high relevance as material system combination for R2R upscaling of indoor OPV devices.

## Materials and methods

Poly[(thiophene)-*alt*-(6,7-difluoro-2-(2-hexyldecyloxy)quinoxaline)] (PTQ10) was purchased from Brilliant Matters with a weight average molecular weight (*M*_w_) and a number average molecular weight (*M*_n_) of 63 and 23 kDa, respectively. [6,6]-Phenyl-C_61_-butyric acid methyl ester (PC_61_BM) was obtained from Solenne BV. *o*-Xylene, diphenyl ether (DPE) and methanol were purchased from Merck (Sigma-Aldrich, for synthesis) and used as received. Prepatterned ITO substrates (100 nm thick) were obtained from Ossila. Commercial ETL ink formulations were purchased from either Avantama (N-10, N-31, N-21X-Flex) or infinityPV, stored in air and used as received (excepting the infinityPV ZnO 5.6% w/v, which was further diluted with 2-propanol in a 1 : 1 (v/v) ratio). The polyethyleneimine-Zn (PEI-Zn) ETL formulation was prepared by dissolving 75 mg of zinc acetate dihydrate (Sigma-Aldrich) onto 1 mL of a 0.1 wt% solution of branched PEI (Sigma-Aldrich, average *M*_w_ 25 kDa, average *M*_n_ 10 kDa) in methanol. This results in an optimized Zn-to-N ratio of 15 : 1 (w/w) as reported elsewhere.^30,31^

All glass-supported devices were prepared in inverted structure as follows. First, ITO substrates were subsequently cleaned and sonicated in acetone, Hellmanex 10 vol% solution in water, 2-propanol and 10 vol% aqueous NaOH solution. Then, the reference ZnO ETL (Avantama N-10) with homogeneous thickness was blade coated (using a ZAA 2300 blade coater and a ZUA 2000 applicator by Zehntner) in air at 5 mm s^−1^ and 40 °C, followed by annealing at 110 °C for 10 minutes prior to being transferred to a nitrogen-filled glovebox. PEI-Zn with homogeneous thickness was deposited following the same parameters as N-10 (in air, at 5 mm s^−1^ and 40 °C); after coating, the annealing temperature gradient on the PEI-Zn layer was performed in air using a Kofler heating bench for a period of 10 minutes. The remaining ETLs were deposited in the glovebox by decelerated blade coating (either from 90 to 5 mm s^−1^; from 30 to 1 mm s^−1^; or from 10 to 1 mm s^−1^) at 60 °C (or 40 °C, for infinityPV ZnO and PEI-Zn) followed by their corresponding annealing treatments. N-31, N-21X-Flex and infinityPV ZnO films were annealed in the glovebox for a period of 5 minutes at 80 °C right after deposition. The best performing PEI-Zn ETL required annealing for 10 minutes at 130 °C in air. Then, the PAL ink at a typical total concentration of 30–50 g L^−1^ was blade coated in the glovebox, either through deceleration (from 90 to 10 mm s^−1^) to obtain a steep thickness gradient or at constant speed (7 mm s^−1^ to obtain PAL thicknesses between 80–100 nm; or 25 mm s^−1^ for layers between 350–200 nm thick). The blade coating temperature was set to 80 °C and the ink vial pre-heated and stirred on a hot plate (80 °C) for at least 3 hours; the vial was exclusively removed from the hot plate for pipetting and deposition. Then, the PAL was annealed for 2 minutes in the glovebox at 80 °C. Finally, 40 nm of MoO_3_ and 120 nm of Ag were thermally evaporated in ultra-high vacuum at a rate of 0.1 and 1 Å s^−1^, respectively. The devices were not encapsulated at any stage.

The manufacture of laminated devices started by slot-die coating (Solar X3, FOM Technologies) a PEDOT:PSS (Clevios PH1000 from Heraeus GmbH) layer on 125-μm-thick PET Melinex ST505 (Tekra) foils. The pristine PH1000 was doped with ethylene glycol (EG, from Sigma-Aldrich) to increase its conductivity, and its wettability improved by adding a nonionic fluorosurfactant (Capstone FS-30, from Dupont) according to the volume ratios 93.5 : 6 : 0.5 (PH1000 : EG : FS-30). The resulting ink was further diluted in distilled water as 2 : 1 (v/v, ink : water). After the coating, the PEDOT layers were baked at 130 °C for 15 minutes. On the cathode side stack, N-31 was used as received and blade coated at 5 mm s^−1^ at 80 °C, while leaving a gap of 75 μm between the wetting edge of the applicator and the PET substrate. The resulting layer was annealed for 2 minutes at 115 °C in air. The PALs on both the cathode and anode stacks were blade coated at 15 mm s^−1^ and 80 °C, followed by an annealing step of 2 minutes in air at 80 °C. The PAL ink was prepared as PTQ10:PC_61_BM 1 : 1.5 (w/w), with a total solid concentration of 40 g L^−1^ in *o*-xylene : DPE (85 : 15, v/v), and the ink was pre-heated at 80 °C before blade coating. The resulting stacks were prepatterned with a scalpel to electrically isolate three different active areas per substrate. Then, the stacks were laminated using a roll laminator (GSS DH-650S Graphical Solutions Scandinavia AB) with a roll temperature of 115 °C and a force of *ca.* 50 N (as measured with a force sensor FlexiForce A201, Tekscan). The final device active areas were defined by the use of apertures painted black to avoid undesirable back reflections during the acquisition of the *J*–*V* curves, which are known to introduce artifacts during their characterization.^57^ The apertures are of two different sizes: 0.25 and 0.07 cm^2^. Finally, two glass slides acting as mechanical support to facilitate the manipulation of the devices were placed at the outer surfaces of the PET foils, thus completing the device structure. Silver paint (Agar AGG302) was added onto the contact areas to reduce the resistance of the PEDOT:PSS. Note that these devices were not encapsulated at any stage. The initial performance of the laminated devices (Table S3†) was first characterized in air under 500 lux (Fig. S19†), and then transferred to a nitrogen-filled glovebox for stability testing. The devices for thermal stability testing are periodically took out from the oven at 70 °C in the glovebox and measured in air, guaranteeing that thermalization is reached before *J*–*V* curve acquisition. Those devices subjected to photostability testing were introduced in an oven at 55 °C in the glovebox for a period of 24 hours before exposure to continuous illumination, and their *J*–*V* curves periodically measured in the glovebox.

The *J*–*V* curves of glass-supported samples were automatically acquired in air using a Keithley 2400 source meter in combination with an Arduino-based multiplexer/switcher that allows data collection of up to 24 devices in a row. A SAN-EI Electric, XES-100S1 AAA solar simulator was used as AM1.5G illumination source. The solar simulator was previously calibrated with a certified silicon solar cell (NREL). For controlling the illumination intensity, different metallic filters with holes drilled were placed in the dedicated slots of the solar simulator. These filters guarantee a flat spectral response upon attenuation. The attenuated irradiance was measured using the abovementioned calibrated and certified silicon solar cell. For indoor LED illumination conditions of glass-supported samples, a SINUS-70 (WAVELABS Solar Metrology Systems GmbH) LED solar simulator was employed. For laminated devices, the *J*–*V* curves were acquired using a Keithley 2400 source meter in combination with a customized indoor LED simulation black box. The LED emission spectrum was collected with a QE-Pro (Ocean Optics) spectrometer and the light intensity calibrated with a Hamamatsu Si photodiode S1133-01.

For measuring PAL thicknesses, we gently removed the PAL in between the active pixel areas using neat *o*-xylene, which was found to be an orthogonal solvent for all the ETLs tested. This procedure left exposed edges of the PAL and ETLs that could be measured by means of a KLA Tencor D-500 profilometer.

Spectroscopic ellipsometry data of blade coated PTQ10:PC_61_BM films were acquired with a dual rotating compensator ellipsometer (RC2, J. A. Woollam Co., Inc.) at seven angles of incidence in the range 45°–75°. Ellipsometry data were analysed with CompleteEASE (J. A. Woollam Co., Inc.) in a three-phase model (substrate/layer/ambient) using non-linear regression methods whereby the thickness of a layer and its optical function were fitted. Kramers–Kronig consistent B-splines were used to model layer optical functions. The results were invariant under sample rotation indicating negligible in-plane anisotropy.

The prediction of upper limits for photocurrent generation in devices was done with optical models of materials in a device stack as follows: semi-infinite air; glass (incoherent transmission); ITO (100 nm); ETL (35 nm); PAL (variable thickness); MoO_3_ (40 nm); and Ag (120 nm). The calculation, based on transfer-matrix modelling,^58^ was performed using custom code written on Python and using NumPy.^59^ For the computation of *J*_sc_, the integration range was set from 355 to 800 nm while using the tabulated AM1.5G irradiance spectrum.

## Conclusions

In this work we have introduced an optimized ink formulation to use as PAL in organic solar cells. The proposed blend includes PTQ10 as donor polymer and PC_61_BM as fullerene acceptor, which is a particularly appealing combination for upscaling due to its inherent low synthetic complexity and the use of non-halogenated co-solvents such as *o*-xylene and diphenyl ether. The PAL shows its maximum performance when thick films (200–400 nm) are employed, which results from the maximized light harvesting and the uncompromised charge transport in the bulk heterojunction. This property is very desirable for future OPV industrialization through R2R, mass-printing methods such as slot-die coating. Then, the PAL is tested with up to five different inorganic ETLs in inverted device architectures, including commercial ZnO, SnO_2_ and Al:ZnO formulations; and a reformulated methanol-based PEI-Zn interlayer. Under simulated 1 sun conditions, the optimized PEI-Zn ETL achieves a maximum PCE of 7.5%, which constitutes a new PCE record for OPV blend systems with a synthetic facility above 80%. PEI-Zn interlayers can further accommodate thicknesses up to 150 nm while keeping FFs above 70%, which renders them particularly relevant for upscaling. For indoor photovoltaic applications, the PAL shows equally promising results with a champion 19.9% PCE. In this scenario, SnO_2_ formulations are superior to Zn-based ETLs as they do not require UV-induced photoactivation to fully unlock their conducting properties. Accordingly, we demonstrate laminated devices for indoor photovoltaic purposes that are manufactured in a way congruent with R2R methods. These air-processed devices show decent thermal- and photostability data, retaining more than 80% of their initial PCE after 400 and 350 hours of testing, respectively. With all that, our non-halogenated PAL ink formulation shows versatility for both outdoor and indoor illumination environments, which positions PTQ10:PC_61_BM blends, PEI-Zn and SnO_2_ interlayers as strong candidates for a more environmentally friendly upscaling of multipurpose organic solar cells in the years to come.

## Conflicts of interest

O. I. and T. O. are co-founders of the company Epishine AB focused on commercializing OPV for indoor applications.

## Supplementary Material

TA-010-D2TA01205G-s001
