# Supplementary material for: Matching electron transport layers with a non-halogenated and low synthetic complexity polymer:fullerene blend for efficient outdoor and indoor organic photovoltaics
Source: J Mater Chem A Mater. 2022 Apr 19;10(19):10768–79. doi: 10.1039/d2ta01205g (PMC9113214; doi:10.1039/d2ta01205g)
Supplement: TA-010-D2TA01205G-s001 [file TA-010-D2TA01205G-s001.pdf]

Electronic Supplementary Material (ESI) for Journal of Materials Chemistry A.
This journal is © The Royal Society of Chemistry 2022

Matching electron transport layers with a non-halogenated and low synthetic complexity polymer:fullerene blend for efficient outdoor and indoor organic photovoltaics

Xabier Rodríguez-Martínez[1], Sergi Riera-Galindo[1,], Jiayan Cong[1], Thomas Österberg[2], Mariano Campoy-Quiles[3], Olle Inganäs[1]*

[1] *Biomolecular and Organic Electronics, Department of Physics, Chemistry and Biology, Linköping University, Linköping, 581 83 Sweden*
[2] *Epishine AB, Wahlbecksgatan 25, Linköping, 582 13 Sweden*
[3] *Instituto de Ciencia de Materiales de Barcelona, ICMAB-CSIC, Campus UAB, Bellaterra, 08193 Spain*

* *Author to whom correspondence should be addressed: sergi.riera-galindo@liu.se*

Supplementary Information

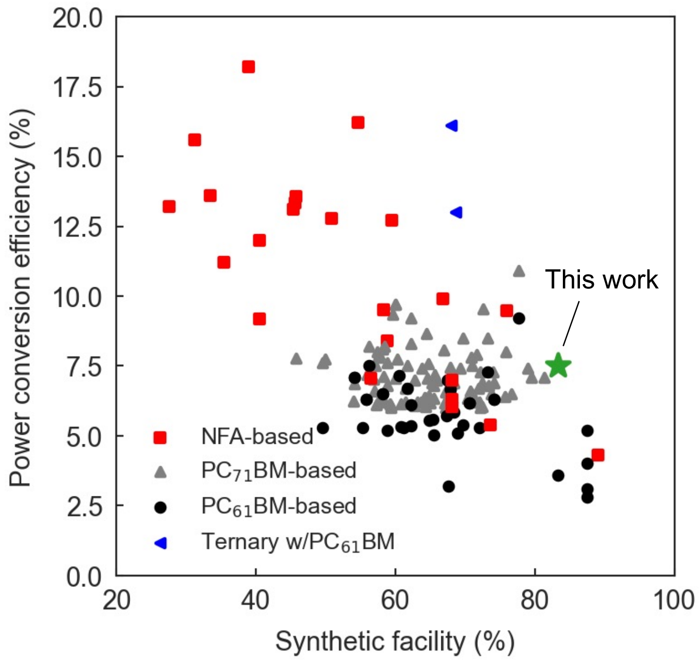

Figure S1. Best power conversion efficiency (PCE) reported for binary and ternary BHJ devices as a function of the synthetic facility of the active layer components. The synthetic facility (SF, %) is computed as 100 – SC (%), SC being the synthetic complexity as defined by Po et al.[1]

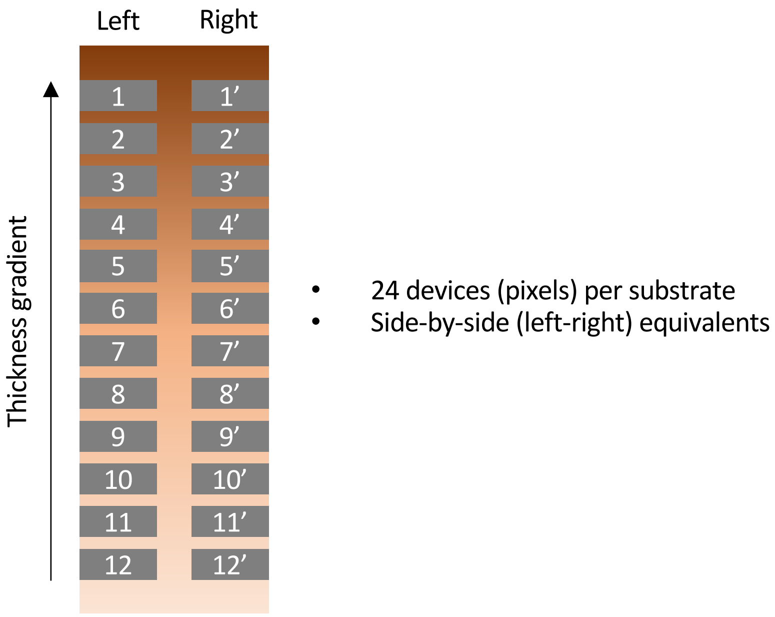

Figure S2. Array of 24 devices (pixels) arranged on a large aspect-ratio, pre-patterned ITO substrate. A PAL thickness gradient is illustrated over the long axis of the substrate. As per the current high-throughput array design and gradient orientation, left- and right-side pixels are equivalent, hence 12 different parametric combinations (with two replicas each) are probed per substrate.

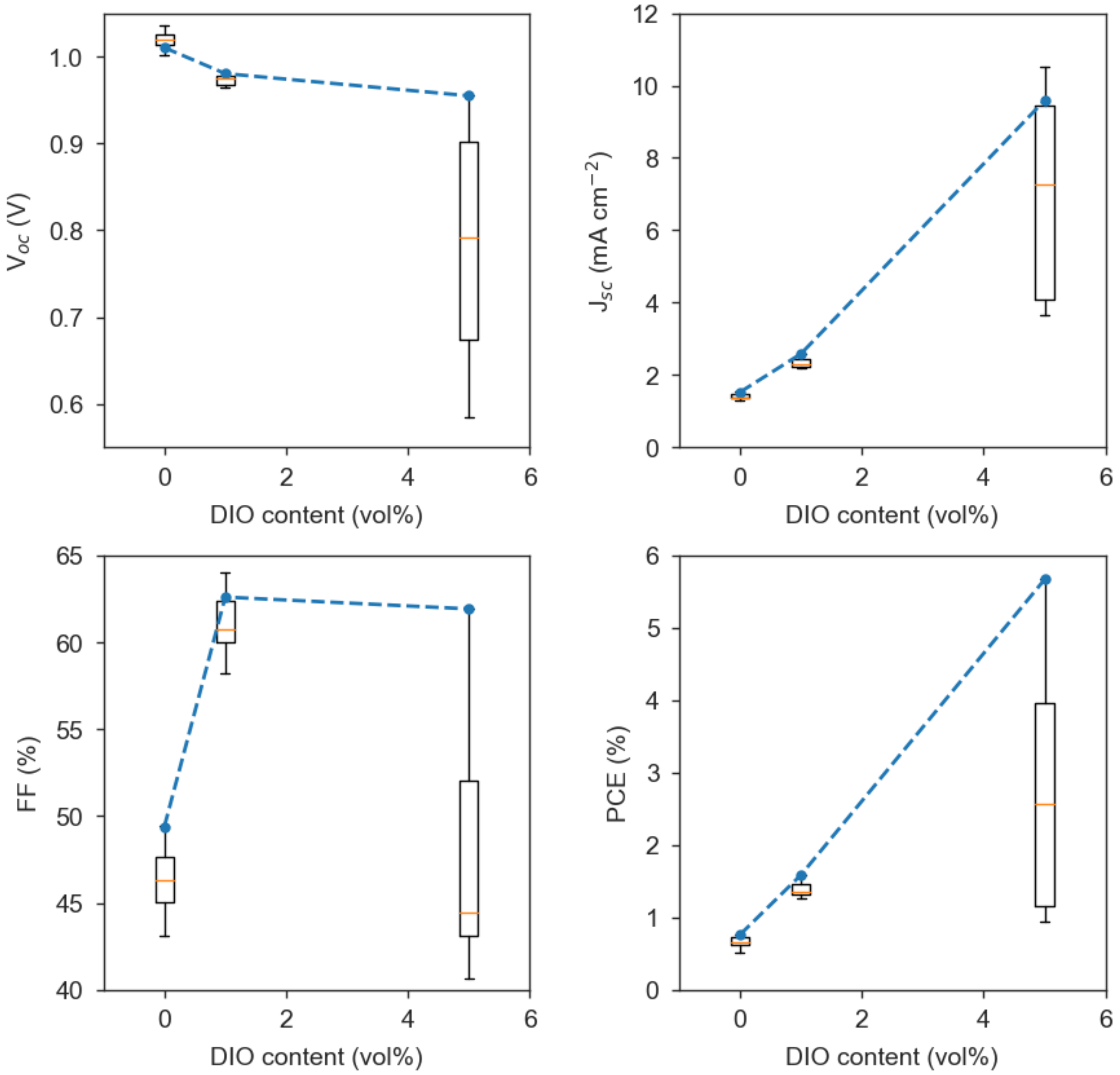

Figure S3. Figures-of-merit (V_{oc}, J_{sc}, FF and PCE) obtained in the screening of DIO vol% in the PTQ10:$PC_{61}BM$ ink formulation using o-xylene as main solvent. Whisker plots represent the dispersion of data obtained in a single substrate with an active layer thickness gradient including 24 individual pixels (devices). The dashed lines connect the datapoints corresponding to the maximum PCE reached at each step of additive content. A total of 96 devices are considered in this plot.

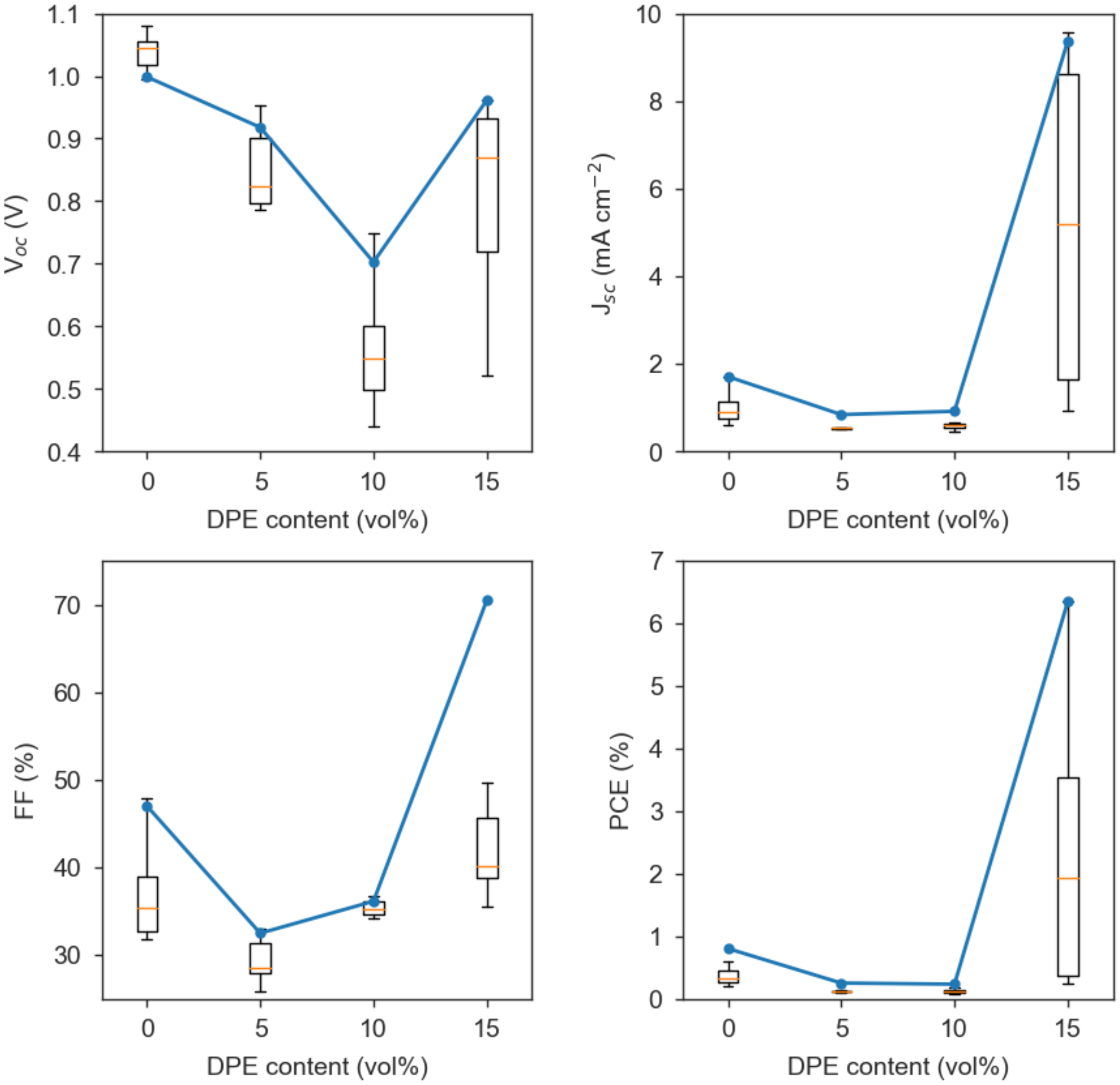

Figure S4. Figures-of-merit (V_{oc}, J_{sc}, FF and PCE) obtained in the screening of DPE vol% in the PTQ10:PC_{61}BM ink formulation using o-xylene as main solvent. Whisker plots represent the dispersion of data obtained in a single substrate with an active layer thickness gradient including 24 individual pixels (devices). The solid lines connect the datapoints corresponding to the maximum PCE reached at each step of additive content. A total of 120 devices are considered in this plot.

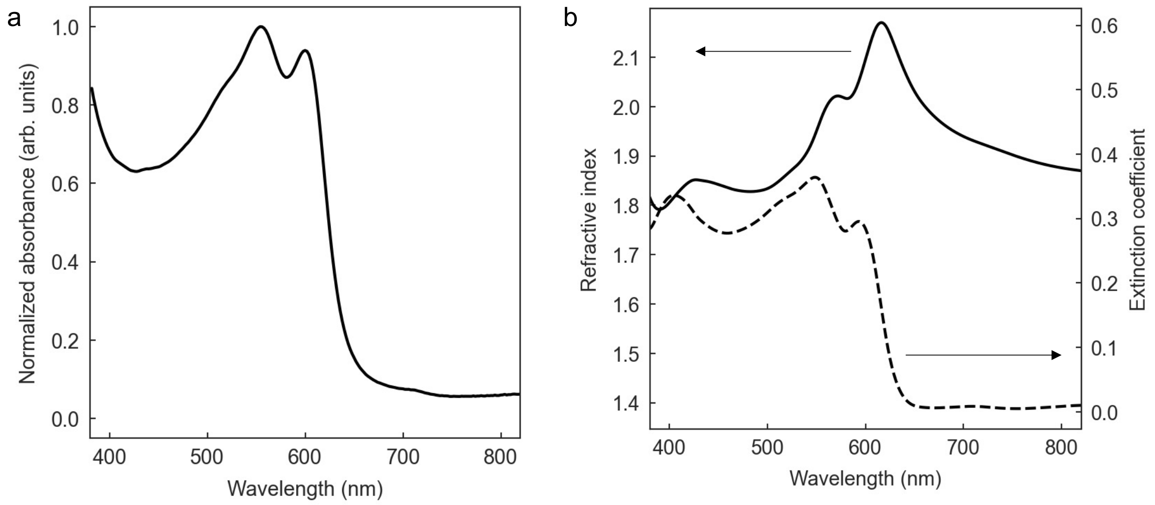

Figure S5. (a) Normalized absorbance of a blade coated PTQ10:$PC_{61}BM$ film with 15 vol% DPE. (b) Complex refractive index of the same film obtained by means of variable-angle spectroscopic ellipsometry (VASE). The refractive index and the extinction coefficient are indicated as solid and dashed lines, respectively.

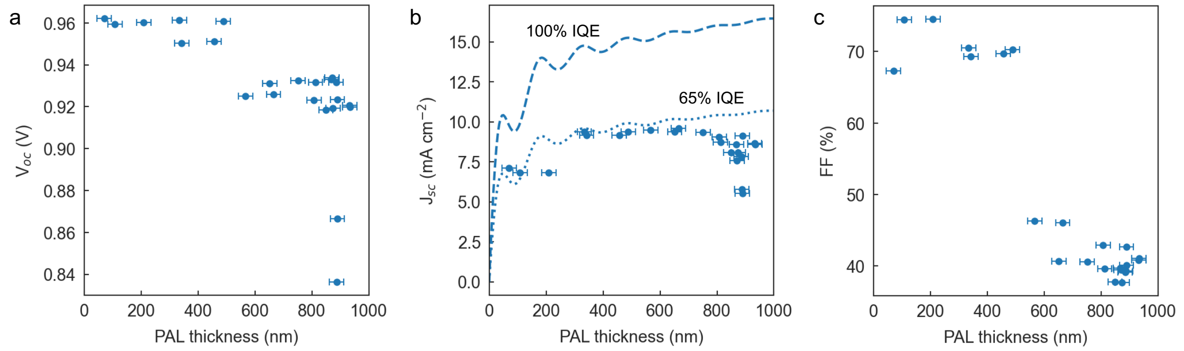

Figure S6. V_{oc} (a), J_{sc} (b) and FF (c) of OPV devices casted from a 15 vol% DPE PAL ink formulation and using a 30-35 nm thick film of N-10 (ZnO) as ETL. The J_{sc} subplot (panel b) includes as dashed and dotted lines the expected photocurrent density as a function of PAL thickness assuming either 100% or 65% internal quantum efficiency (IQE), respectively, as per the results of transfer matrix optical modelling.

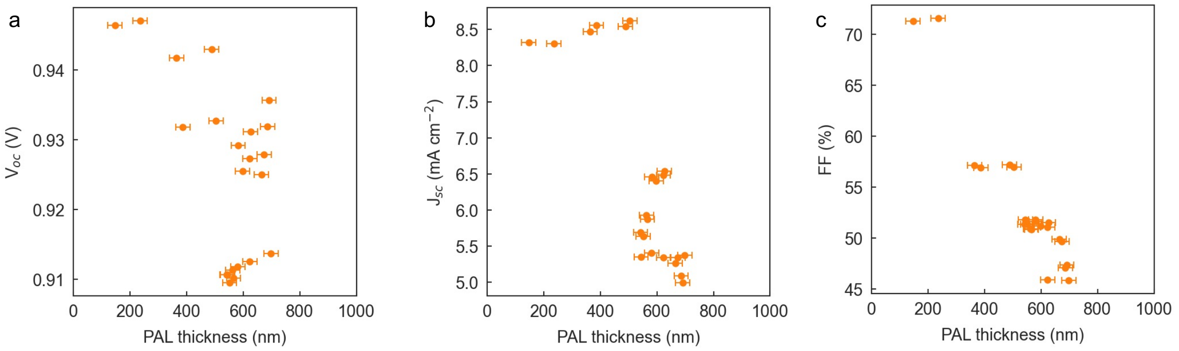

Figure S7. Figures-of-merit of OPV devices casted from a 15 vol% DPE PAL ink formulation and using a 30-35 nm thick film of N-31 (SnO_2) as ETL.

Synthetic complexity and synthetic facility

The synthetic complexity (SC) is a metric introduced by Po et al. in 2015[1] to quantify the chemical complexity and experimental effort required in the synthesis of donor and acceptor materials for organic photovoltaics. SC proposes weighting the number of synthetic steps (NSS), the reciprocal yield (RY), the number of operation units for the isolation/purification (NUO), the number of column chromatographies for the isolation/purification (NCC) and the number of hazardous chemicals (NHC) used in the synthesis of the raw active layer materials according to the following equation:

$$SC\ (\%) = 35\frac{NSS}{NSS_{max}} + 25\frac{\log RY}{\log RY_{max}} + 15\frac{NUO}{NUO_{max}} + 15\frac{NCC}{NCC_{max}} + 10\frac{NHC}{NHC_{max}},$$

where NSS_{max}, RY_{max}, NUO_{max}, NCC_{max} and NHC_{max} correspond to normalization values applied to either donors or acceptors. The figures for normalization initially proposed by Po and co-workers (NSS_{max} = 22; RY_{max} = 86.9; NUO_{max} = 39; NCC_{max} = 13; NHC_{max} = 44) have been recently updated by Chochos et al.[2] to account for the continuous development of the field with novel donor co-polymers and non-fullerene acceptors (NSS_{max} = 20; RY_{max} = 104.3; NUO_{max} = 29; NCC_{max} = 9; NHC_{max} = 52). The normalization values used in this work are shown in Table S1 in agreement with our extensive literature search. Figure 1 in the main text includes synthetic facility (SF) values, which are simply calculated as

$$SF\ (\%) = 100 - SC(\%)$$

to further emphasize the search of both larger PCEs and SF figures.

Table S1. Synthetic complexity of the donor and acceptor materials included in Figure 1 in the main text.

Material	NSS	RY	NUO	NCC	NHC	SC (%)	Reference
PTQ10	4	2.33	6	1	8	15.91	3
J71	9	5.01	18	5	15	38.56	3
PTB7-Th	16	13.3	24	7	31	62.64	4
PBDB-T	11	7	18	5	32	46.81	4
PM6	15	19.8	22	7	32	62.62	4
P3HT	3	1.1	4	0	4	7.59	5
PffBT4T-2OD	8	1.4	13	3	22	27.23	5
D18	10	7.3	19	5	40	47.37	5
PV2000	9	5.4	17	1	33	37.43	5
PCPDTBT	9	10.1	19	7	27	47.34	4
PV2300	9	7.7	17	1	33	39.34	5
TPD-3F	11	14.24	18	5	32	50.63	4
P1	16	15	24	7	31	63.29	1
P2	15	14.8	24	6	19	58.17	1
P3	14	4.3	21	6	44	53.58	1
P4	9	5.5	17	2	19	35.99	1

**P5**	9	7.7	17	5	29	43.18	[1]
**P6**	14	7.1	26	8	32	58.20	[1]
**P7**	18	10.9	30	9	35	70.14	[1]
**P8**	15	18.1	25	11	21	65.79	[1]
**P9**	14	14.5	28	11	31	66.08	[1]
**P10**	12	10	22	4	21	48.59	[1]
**P11**	7	7.2	10	2	15	30.79	[1]
**P12**	16	18	27	9	31	67.73	[1]
**P13**	10	5.1	14	4	24	39.29	[1]
**P14**	14	22.2	29	11	23	67.22	[1]
**P15**	17	8	27	10	38	67.46	[1]
**P16**	9	5	17	5	29	40.86	[1]
**P17**	22	36.5	37	13	39	91.08	[1]
**P18**	19	86.9	39	9	16	82.71	[1]
**P19**	14	37	23	7	18	62.08	[1]
**P20**	21	21.1	36	13	25	83.47	[1]
**P21**	15	21.7	26	9	21	64.84	[1]
**P22**	12	18.1	18	5	30	53.13	[1]
**P23**	10	5.7	19	4	18	40.66	[1]
**P24**	13	25	26	6	22	59.15	[1]
**P25**	18	19	28	9	14	68.32	[1]
**P26**	11	6	21	3	28	44.06	[1]
**P27**	5	4	10	3	10	24.64	[1]
**P28**	13	10.4	24	5	34	54.82	[1]
**P29**	7	5.8	15	4	19	34.63	[1]
**P30**	8	7.9	11	5	23	38.27	[1]
**P31**	11	20.5	19	3	35	51.25	[1]
**P32**	13	64.4	24	10	35	70.59	[1]
**P33**	15	30	24	6	39	65.81	[1]
**P34**	5	4.4	8	2	12	23.62	[1]
**P35**	5	3.1	6	1	12	19.81	[1]
**P36**	15	57.1	30	9	35	74.28	[1]
**P37**	12	9.2	24	7	26	53.34	[1]
**P38**	14	21.7	24	6	39	62.48	[1]
**P39**	14	37.9	24	9	38	68.75	[1]
**P40**	12	8.9	22	7	17	50.66	[1]
**P41**	13	11	23	7	34	57.04	[1]
**P42**	15	4.1	11	5	32	47.61	[1]
**P43**	12	4.5	22	9	31	51.99	[1]
**P44**	9	7.1	15	3	18	37.55	[1]
**P45**	8	26.9	14	5	22	45.82	[1]
**P46**	8	5.3	13	3	22	34.39	[1]

**P47**	17	25.8	30	12	23	74.34	1
**P48**	16	10.3	30	7	38	64.92	1
**P49**	13	7.2	20	6	29	51.49	1
**P50**	9	5.6	17	2	18	35.89	1
**P51**	15	7.3	30	7	29	59.75	1
**P52**	8	7.3	14	5	21	38.61	1
**P53**	11	7	18	5	32	46.81	1
**P54**	14	13.6	33	12	29	68.43	1
**P55**	10	6	16	3	31	41.12	1
**P56**	11	18	18	4	18	48.05	1
**P57**	17	12	31	6	36	66.18	1
**P58**	10	8.2	19	3	13	40.50	1
**P59**	7	7.4	12	5	14	34.98	1
**P60**	5	14.2	6	2	13	29.34	1
**P61**	3	1.1	4	0	4	7.59	1
**P62**	11	9.5	23	8	27	52.88	1
**P63**	7	4.4	12	3	21	31.22	1
**P64**	13	4.7	23	7	38	53.24	1
**P65**	14	6.8	23	7	24	54.12	1
**P66**	8	17	17	4	14	41.81	1
**P67**	13	7.8	22	4	35	51.54	1
**P68**	18	11.6	30	11	25	70.86	1
**P69**	9	3.1	12	4	24	34.25	1
**P70**	15	37.2	23	8	30	67.16	1
**P71**	17	60	31	7	29	74.65	1
**P72**	9	8	16	4	21	40.31	1
**P73**	14	17	21	5	43	59.63	1
**P74**	16	13.2	31	8	27	65.68	1
**P75**	13	18	25	5	18	55.08	1
**P76**	10	9.8	17	3	12	40.49	1
**P77**	14	8.6	23	5	41	56.35	1
**P78**	11	7.4	20	7	19	47.69	1
**P79**	10	3.2	17	6	15	38.51	1
**P80**	10	10.1	21	7	42	52.58	1
**P81**	10	5.7	17	3	31	41.23	1
**P82**	12	9.2	23	5	14	48.34	1
**P83**	14	10.3	25	7	44	60.97	1
**P84**	16	21.7	25	8	17	64.12	1
**P85**	16	16.3	27	9	35	67.97	1
**P86**	15	11.4	29	10	22	63.88	1
**P87**	14	6.9	23	7	39	57.09	1
**P88**	8	5.2	15	5	24	37.75	1

P89	12	9.7	24	9	16	54.01	[1]
P90	11	7.9	18	6	30	48.23	[1]
P91	9	4.5	19	4	20	38.18	[1]
P92	14	19.4	25	8	35	63.80	[1]
PB	9	5.6	17	2	19	36.09	[1]
PC	15	11.9	28	6	35	61.61	[1]
PL	15	14.8	24	6	19	58.17	[1]
PM	10	8.2	21	4	30	45.69	[1]
PN	12	7.4	24	4	22	47.93	[1]
PO	13	8.6	21	6	24	51.87	[1]
PQ	9	8.8	19	6	15	43.13	[1]
PR	15	7.3	29	5	35	58.21	[1]
PU	16	20.4	33	12	20	72.06	[1]
PV	9	6.2	16	3	24	38.36	[1]
PW	14	14.3	27	6	33	60.24	[1]
PY	11	6.1	19	5	23	44.73	[1]
PZ	13	8.3	21	8	11	51.49	[1]
PQSi05	7.05	3	13.05	1.05	14.1	26.07	[6]
PQSi10	7.1	3.01	13.1	1.1	14.2	26.26	[6]
PQSi25	7.25	3.04	13.25	1.25	14.5	26.84	[6]
Normalization	22	104.3	39	13	52	N/A	[1,2]

Material	NSS	RY	NUO	NCC	NHC	SC (%)	Reference
$PC_{61}BM$	4	1.9	0	2	8	17.41	[3]
4TIC-4F	13	4.4	16	7	47	66.26	[3]
Y6	15	8.9	28	6	25	74.78	[4]
ID4F	15	48.31	24	9	32	90.10	[3]
$PC_{71}BM$	4	1.9	0	2	8	17.41	[3]
O-IDTBR	11	3.3	24	6	16	56.16	[5]
ITIC-4F	13	44.5	22	8	30	82.33	[5]
ZY-4Cl	6	6.6	7	5	19	40.52	[5]
PV-A3	7	5.2	11	2	24	39.17	[5]
EH-IDTBR	11	3.3	24	6	16	56.16	[5]
N2200	8	7.62	4	1	33	40.32	[4]
IDIC	13	5.56	24	6	23	65.13	[6]
ITIC	10	4	22	6	17	54.52	[6]
ITIC-4Cl	10	2.5	20	7	16	51.91	[6]
BTP-4Cl	17	20	29	6	27	85.06	[6]
A1	7	3.5	16	5	29	45.27	[7]
3PS2-SiPc	2	3.9	1	0	4	14.26	[8]
Normalization	17	48.31	29	9	47	N/A	[6]

Table S2. Characteristics of the commercial and pristine ETL ink formulations used in this work (IPA = 2-propanol). Unless otherwise specified, all information is collected from the corresponding product brochures provided by the manufacturers. Note that in this study the pristine infinityPV ZnO formulation is further diluted with 2-propanol (50 vol%) to half its solid content and match it with the remaining ETL formulations.

Name	Supplier	Material	Particle size (nm)	Work function (eV)	Concentration	Solvent	Viscosity (cP)
N-10	Avantama	ZnO	12	4.3	2.5 wt%	IPA	2.4
N-31	Avantama	SnO_2	7	4.5[9]	2.5 wt%	Butanols	3.5
N-21X-Flex	Avantama	Al:ZnO	12	3.9	2.5 wt%	Butanols	3.5
ZnO	infinityPV	ZnO	N/A	N/A	5.6% w/v	IPA	1.9

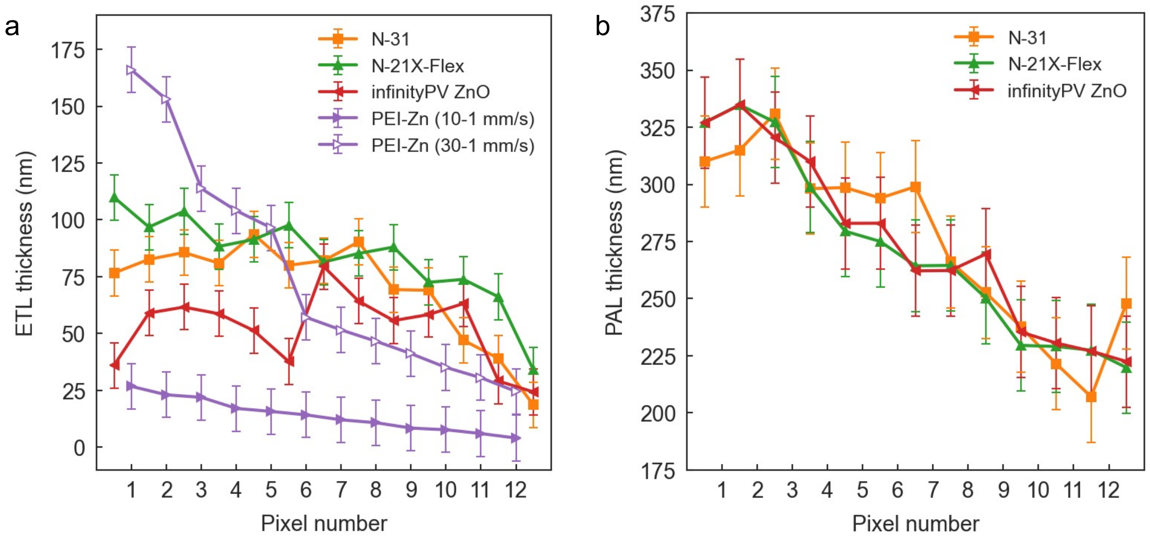

Figure S8. (a) ETL thickness as a function of pixel number resulting from decelerated blade coating (typically from 10 mm s^{-1} to 1 mm s^{-1}) during ETL deposition. (b) PAL thickness as a function of pixel number in those same devices. Note that in this case the PAL thickness is deposited at constant blade speed; with all that, we observe that the PAL thickness decreases along the blade coating direction due to PAL ink depletion at the blade reservoir. This effect is further pronounced due to the use of large blade coating speeds (25 mm s^{-1}) as required to get thick enough PALs (>200 nm) that maximize light harvesting.

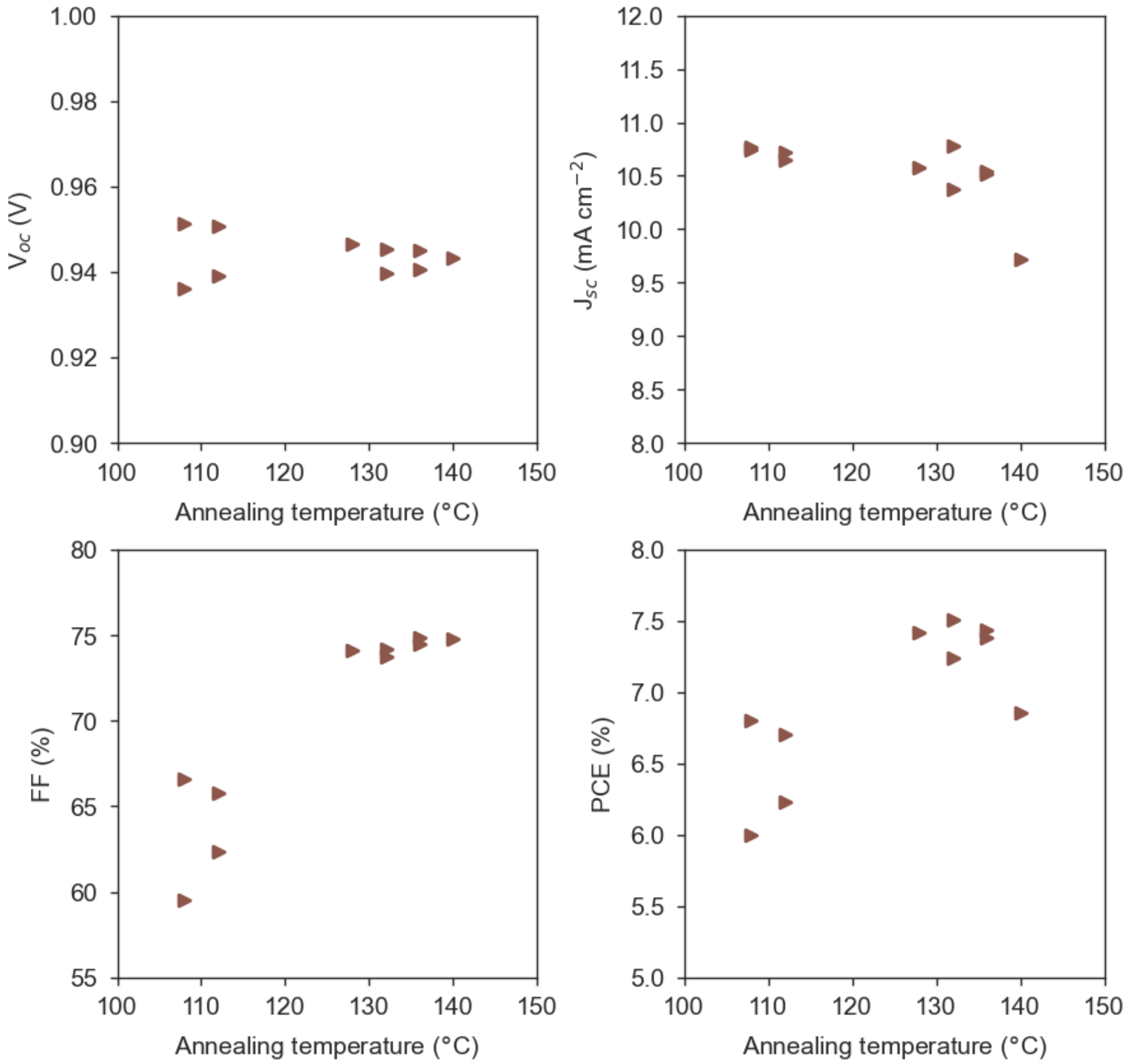

Figure S9. Figures-of-merit (V_{oc}, J_{sc}, FF and PCE) obtained in the optimization of the annealing temperature of a homogeneous PEI-Zn film as ETL (deposited at 5 mm s^{-1}). The annealing treatment is performed in air for a period of 10 minutes using a Kofler bench comprising temperatures from 104 to 148 ℃. The previously optimized DPE-rich PTQ10:PC_{61}BM ink formulation is employed as active layer (blade coated at a speed of 25 mm s^{-1}).

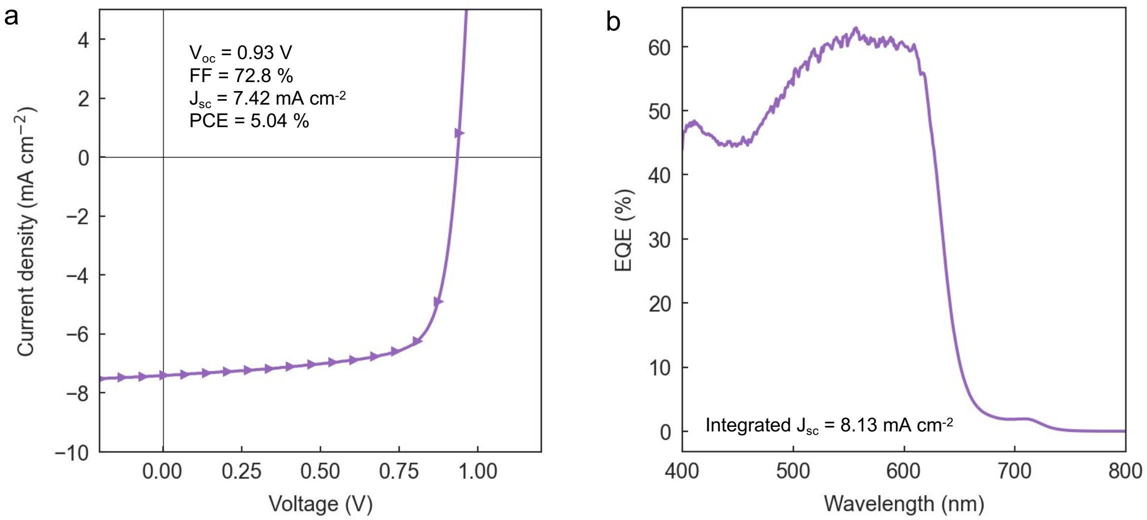

Figure S10. (a) JV curve and the corresponding photovoltaic figures-of-merit of a representative solar cell including PEI-Zn as ETL and PTQ10:PC_{61}BM (15 vol% DPE) as PAL. (b) Its corresponding external quantum efficiency (EQE) and integrated J_{sc} value (integration performed using the AM1.5G irradiance spectrum).

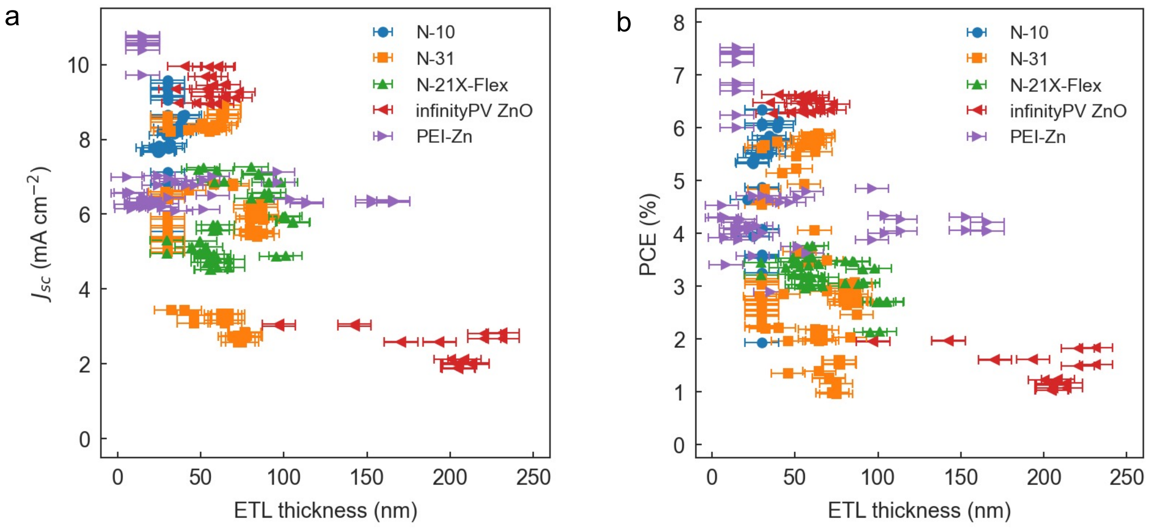

Figure S11. (a) J_{sc} and (b) PCE as a function of ETL thickness for the different interlayers tested in this work.

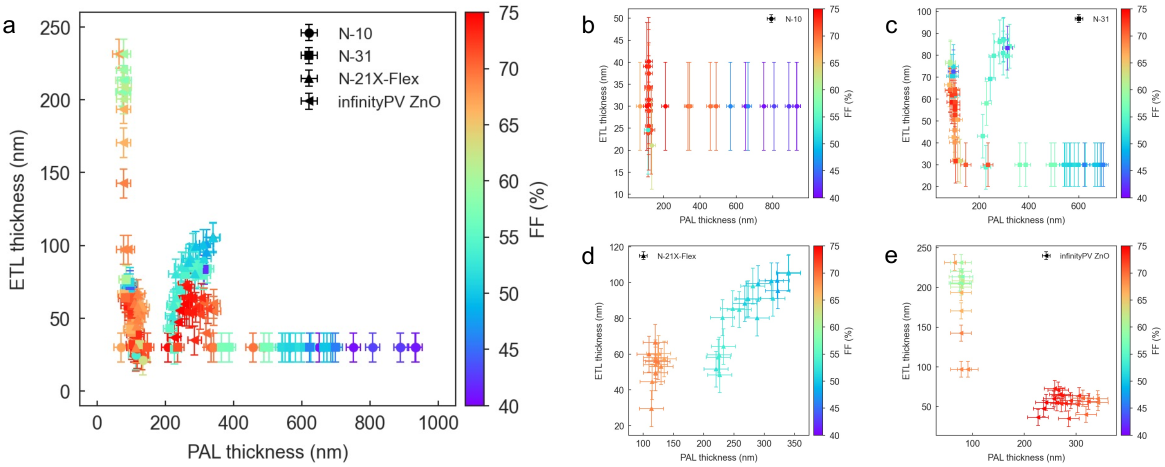

Figure S12. Correlation plots between the ETL thickness, the PAL thickness, and the FF. The FF is quantified with the colour scale. (a) Aggregated data for all available devices. (b) Data for N-10 devices. (c) Data for N-31 devices. (d) Data for N-21X-Flex devices. (e) Data for infinityPV ZnO devices.

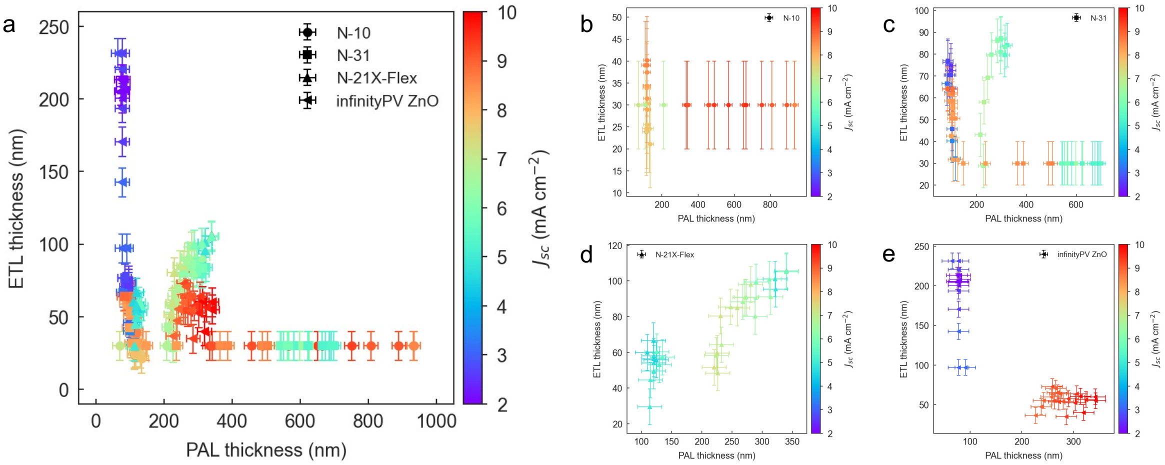

Figure S13. Correlation plots between the ETL thickness, the PAL thickness, and the J_{sc}. The J_{sc} is quantified with the colour scale. (a) Aggregated data for all available devices. (b) Data for N-10 devices. (c) Data for N-31 devices. (d) Data for N-21X-Flex devices. (e) Data for infinityPV ZnO devices.

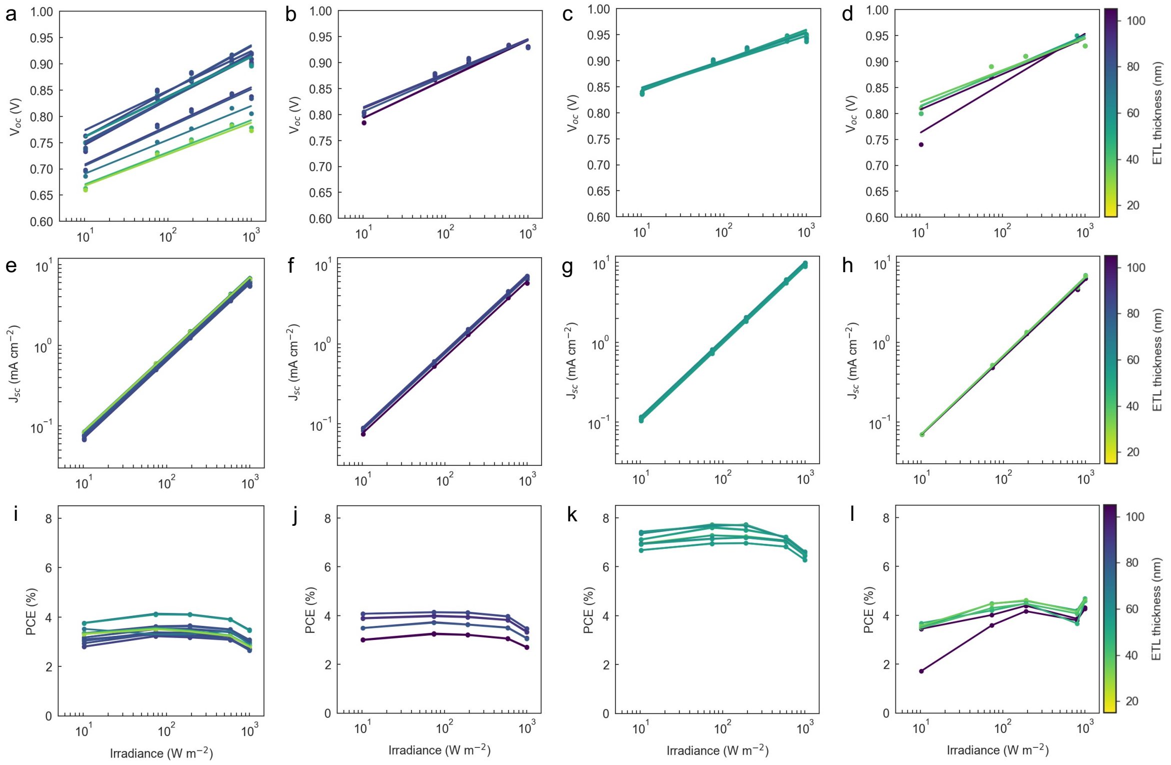

Figure S14. V_{oc}, J_{sc} and PCE as a function of irradiance and ETL thickness for PTQ10:$PC_{61}BM$ devices comprising thick active layers (200-350 nm). Regarding the choice of ETL, panels (a,e,i) correspond to N-31; panels (b,f,j) to N-21X-Flex; panels (c,g,k) to infinityPV ZnO; and panels (d,h,l) to PEI-Zn.

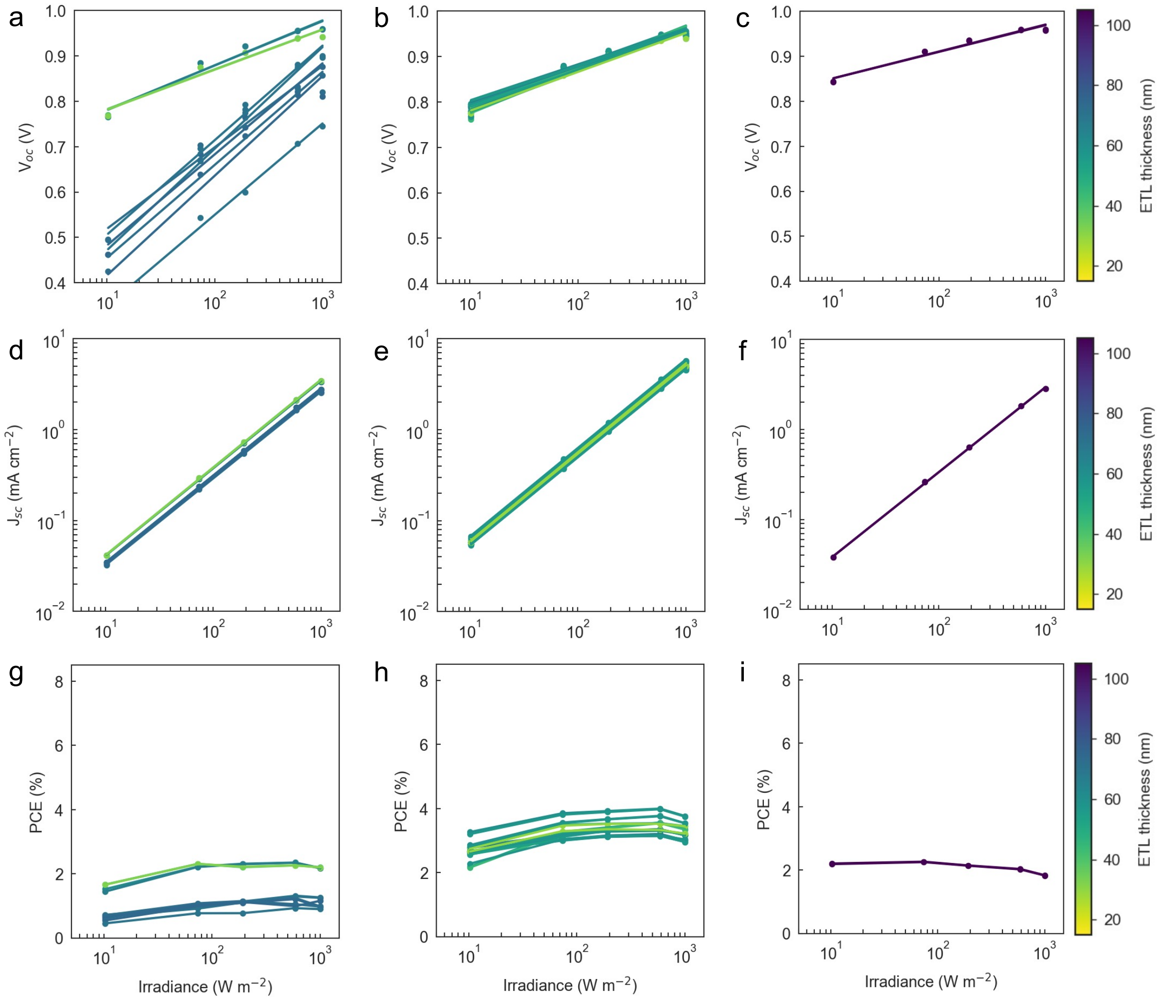

Figure S15. V_{oc}, J_{sc} and PCE as a function of irradiance and ETL thickness for PTQ10:$PC_{61}BM$ devices comprising thin active layers (80-100 nm). Regarding the choice of ETL, panels (a,d,g) correspond to N-31; panels (b,e,h) to N-21X-Flex; and panels (c,f,i) to infinityPV ZnO.

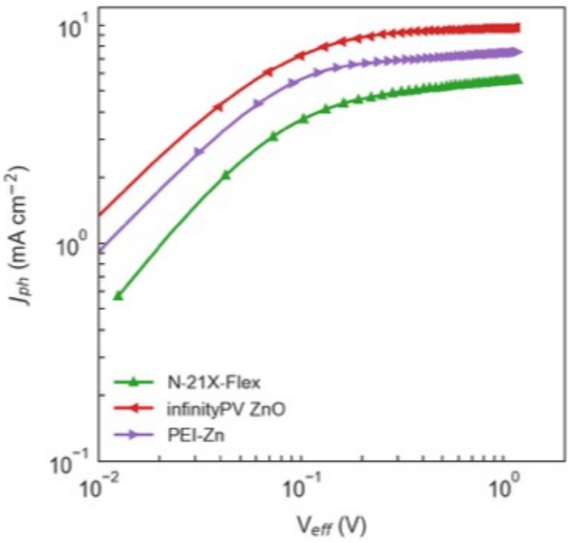

Figure S16. Effective photocurrent density (J_{ph}) as a function of effective voltage (V_{eff}). J_{ph} is defined as $J_{ph} = J_{light} - J_{dark}$, where J_{light} and J_{dark} correspond to the current density of the solar cell under illumination (1 sun) and in dark, respectively. Then, V_{eff} is defined as $V_{eff} = V_0 - V_a$, where V_0 corresponds to the (applied) voltage where $J_{ph} = 0$, and V_a is the applied voltage. Finally, the exciton dissociation efficiency (P_{diss}) and the charge collection efficiency (P_{coll}) are determined as the J_{ph}/J_{sat} values found under short-circuit conditions ($V_a = 0$) and at the maximal power output conditions, being J_{sat} the saturated J_{ph} (i.e., J_{ph} at an effective voltage of 1.1 V in our case).[10,11]

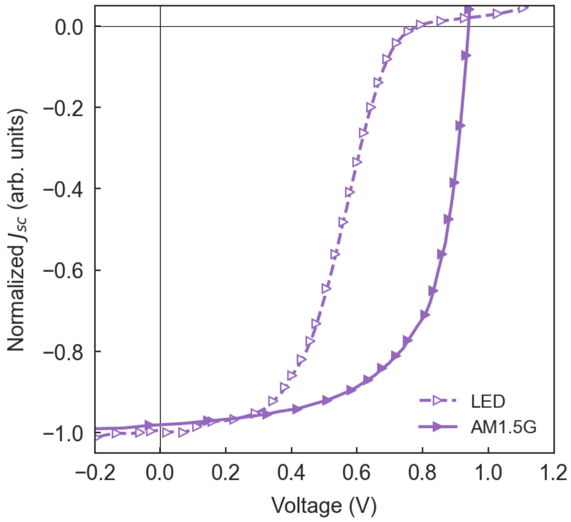

Figure S17. Normalized JV curves of an organic solar cell including PEI-Zn as ETL. Normalization is performed to aid in the identification of the S-shape under LED illumination conditions, as both curves occur in very different current regimes. For the normalization, we divide the corresponding current densities by the value attained under reverse bias (-0.5V), for each illumination condition.

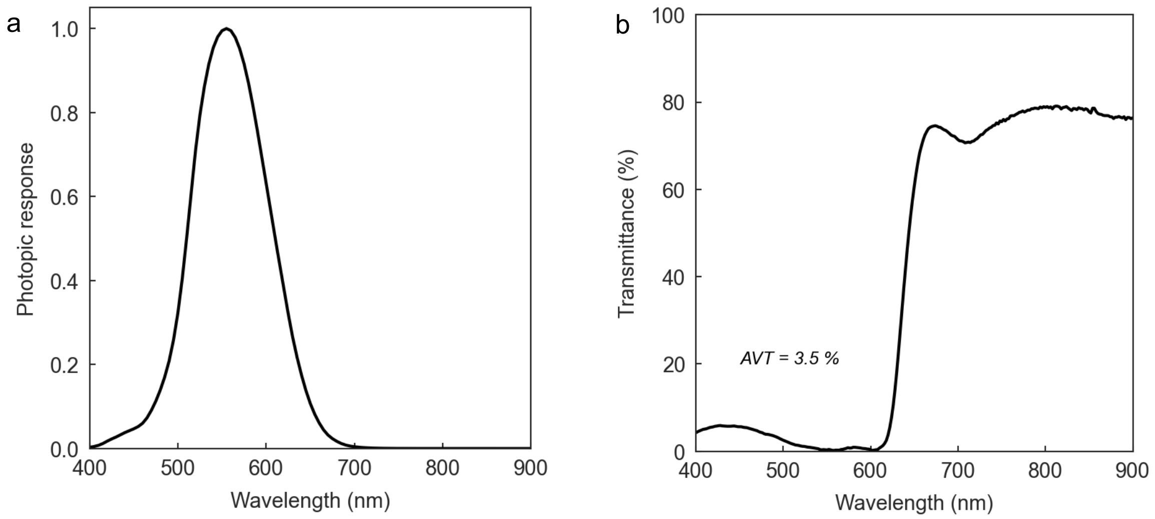

Figure S18. (a) The 1978 CIE photopic luminosity function, $CIE(\lambda)$, as a function of wavelength (λ). (b) Transmittance, $T(\lambda)$, of a laminated device as seen from the cathode side. The average visible transmittance (AVT) is determined as $AVT = \frac{\int T(\lambda)\Phi(\lambda)CIE(\lambda)\,\mathrm{d}\lambda}{\int \Phi(\lambda)CIE(\lambda)\,\mathrm{d}\lambda}$, where $\Phi(\lambda)$ is the AM1.5G irradiance spectrum.

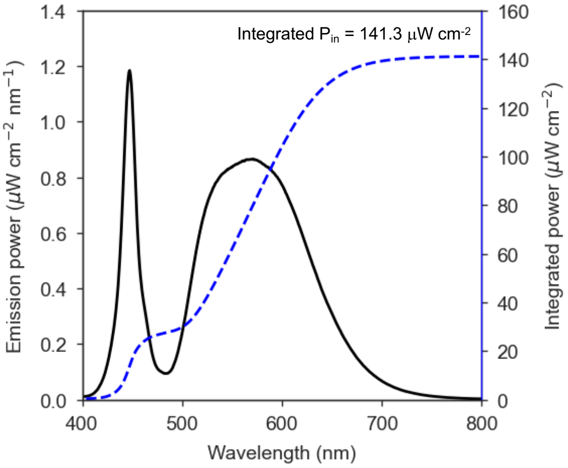

Figure S19. Indoor LED emission power spectrum (solid black line) and integrated input power (P_{in}, blue dashed line) of the light source employed in the photovoltaic characterization of laminated devices.

Table S3. Initial performance of the devices employed in the thermal- (TS) and photostability (PS) studies. Note that these devices were processed entirely in air following R2R-compatible procedures. The illumination source corresponds to the spectrum shown in Figure S19.

Device	Active area (cm^2)	V_{oc} (V)	J_{sc} ($\mu A\ cm^{-2}$)	*FF*	*PCE* (%)
TS-1	0.25	0.75	24.2	69.0	8.8
TS-2	0.07	0.70	30.8	68.5	10.4
TS-3	0.25	0.74	27.8	70.6	10.4
PS-1	0.25	0.72	25.2	70.7	9.0
PS-2	0.07	0.66	30.7	71.5	10.3
PS-3	0.25	0.72	28.0	71.4	10.2
Average	N/A	0.72	28	70	9.8
Standard deviation	N/A	0.03	3	1	0.7

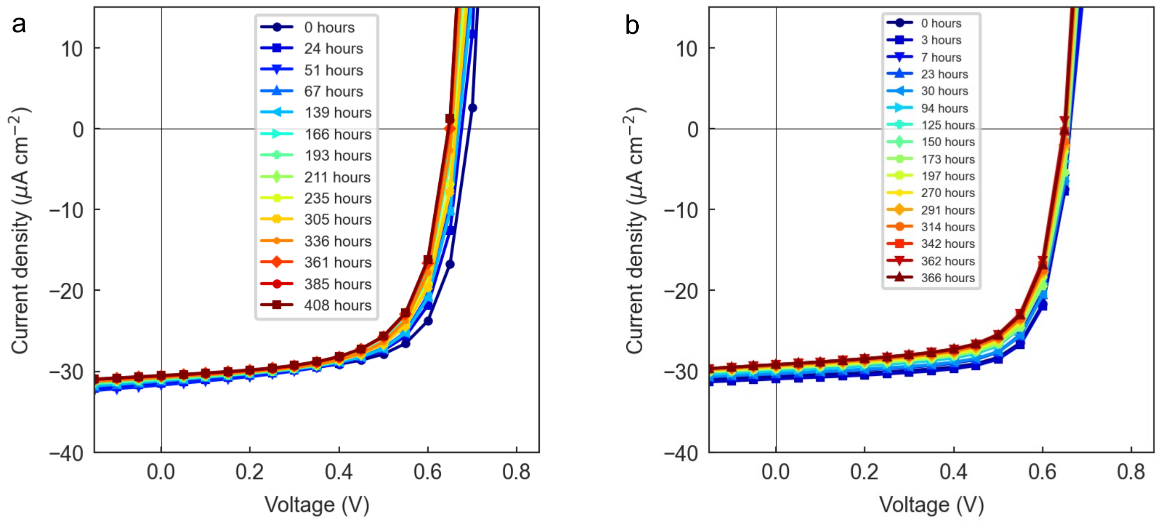

Figure S20. (a) JV curves of the device TS-2 during the thermal stability study. (b) JV curves of the device PS-2 during the photostability study.

References

1 R. Po, G. Bianchi, C. Carbonera and A. Pellegrino, *Macromolecules*, 2015, **48**, 453–461.

2 C. L. Chochos, M. Spanos, A. Katsouras, E. Tatsi, S. Drakopoulou, V. G. Gregoriou and A. Avgeropoulos, *Prog. Polym. Sci.*, 2019, **91**, 51–79.

3 R. Szymanski, R. Henry, S. Stuard, U. Vongsaysy, S. Courtel, L. Vellutini, M. Bertrand, H. Ade, S. Chambon and G. Wantz, *Sol. RRL*, 2020, **4**, 2000538.

4 C. J. Brabec, A. Distler, X. Du, H. Egelhaaf, J. Hauch, T. Heumueller and N. Li, *Adv. Energy Mater.*, 2020, **10**, 2001864.

5 C.-Y. Liao, Y.-T. Hsiao, K.-W. Tsai, N.-W. Teng, W.-L. Li, J.-L. Wu, J.-C. Kao, C.-C. Lee, C.-M. Yang, H.-S. Tan, K.-H. Chung and Y.-M. Chang, *Sol. RRL*, 2021, **5**, 2000749.

6 D. Yuan, F. Pan, L. Zhang, H. Jiang, M. Chen, W. Tang, G. Qin, Y. Cao and J. Chen, *Sol. RRL*, 2020, **4**, 2000062.

7 T. R. Andersen, A. T. Weyhe, Q. Tao, F. Zhao, R. Qin, S. Zhang, H. Chen and D. Yu, *Mater. Adv.*, 2020, **1**, 658–665.

8 T. M. Grant, C. Dindault, N. A. Rice, S. Swaraj and B. H. Lessard, *Mater. Adv.*, 2021, **2**, 2594–2599.

9 I.-G. Bae and B. Park, *Sustain. Energy Fuels*, 2020, **4**, 3115–3128.

10 W. Li, J. Cai, F. Cai, Y. Yan, H. Yi, R. S. Gurney, D. Liu, A. Iraqi and T. Wang, *Nano Energy*, 2018, **44**, 155–163.

11 M. Chen, D. Liu, W. Li, R. S. Gurney, D. Li, J. Cai, E. L. K. Spooner, R. C. Kilbride, J. D. McGettrick, T. M. Watson, Z. Li, R. A. L. Jones, D. G. Lidzey and T. Wang, *ACS Appl. Mater. Interfaces*, 2019, **11**, 26194–26203.
